# Cancer Risk Prediction Using Machine Learning for Supporting Early Cancer Diagnosis in Symptomatic Patients: A Systematic Review of Model Types

**DOI:** 10.1002/cam4.71463

**Published:** 2025-12-13

**Authors:** Flavia Pennisi, Stefania Borlini, Hannah Harrison, Rita Cuciniello, Anna Carole D'Amelio, Matthew Barclay, Giovanni Emanuele Ricciardi, Georgios Lyratzopoulos, Cristina Renzi

**Affiliations:** ^1^ PhD National Programme in One Health Approaches to Infectious Diseases and Life Science Research, Department of Public Health, Experimental and Forensic Medicine University of Pavia Pavia Italy; ^2^ School of Medicine Università Vita‐Salute San Raffaele Milano Italy; ^3^ Department of Public Health and Primary Care, School of Clinical Medicine University of Cambridge Cambridge UK; ^4^ Research Department of Behavioural Science and Health, Institute of Epidemiology and Health Care University College London London UK

**Keywords:** artificial intelligence, cancer, early detection of cancer, machine learning, signs and symptoms

## Abstract

**Introduction:**

Predictive models could support clinicians in identifying patients who may benefit from cancer investigations. We aimed to examine published evidence on machine learning models (ML) developed to estimate cancer risk based on symptoms and other patient characteristics.

**Methods:**

Using MEDLINE, Scopus, and EMBASE, we performed a systematic review of studies published in 2014–2024, which included data on signs/symptoms for cancer risk prediction. We used the QUADAS‐AI tools to assess study quality. We performed a quantitative synthesis of diagnostic performance, including accuracy, sensitivity, specificity, area under the curve (AUC). Adherence to TRIPOD guidelines was assessed.

**Results:**

Among the 5646 initially identified articles, 34 met inclusion criteria. Included studies most frequently examined lung (*n* = 9 studies), mesothelioma (*n* = 7), and gastrointestinal cancers (*n* = 4) and used hospital electronic health records (*n* = 8) or publicly available online datasets (*n* = 13). In addition to signs/symptoms (*n* = 34), most models included sociodemographic characteristics (*n* = 27) and lifestyle factors (*n* = 20). In 70% of studies, internal validation was performed. ML models demonstrated variable performance, with AUC values ranging from 0.60 to 1 during validation. Random Forest, Support Vector Machine, Decision Tree, and Multilayer Perceptron showed the best predictive performance. Most of the studies (94.1%) had a high risk of bias for the index test.

**Conclusion:**

ML models have been reported to demonstrate potential in managing complex data for cancer risk prediction. However, the current evidence is heterogeneous and frequently limited by bias and incomplete reporting. Further validation and thorough assessments of real‐world performance are necessary before these models can be considered reliable for clinical use.

**Trial Registration:**

International Prospective Register of Systematic Reviews (PROSPERO) registration number: CRD42024548088

## 1 Introduction

The majority of cancers are diagnosed after patients present with symptoms, rather than through screening [1, 2, 3, 4, 5, 6]. For example, over 85% of colorectal cancers [1, 2] are diagnosed following symptomatic presentation in the UK, despite the availability of a national colorectal cancer screening programme. The non‐specific nature of many presenting symptoms of as‐yet‐undiagnosed cancer, combined with the frequent presence of non‐neoplastic chronic conditions, complicates the diagnostic process, with clinicians facing significant challenges in determining which patients might benefit from diagnostic investigations [7, 8, 9, 10]. Thus, despite ongoing efforts to diagnose cancer early, a substantial proportion of patients continue to be diagnosed as emergencies and/or at an advanced stage [11, 12, 13]. In the UK, approximately 22% of colorectal cancers and more than 45% of lung cancers are diagnosed following an emergency presentation [14], which is associated with worse outcomes even after adjusting for stage at diagnosis [15, 16].

Advancements in technology, such as the integration of machine learning (ML) in clinical practice, may be opening new avenues to enhance diagnostic processes [17, 18, 19, 20, 21, 22]. ML's capability to analyze complex biomedical data offers unprecedented opportunities to accelerate and refine the accuracy of cancer risk assessment and diagnostic approaches. When patients present with symptoms, ML tools could help clinicians discriminate between those who require urgent investigations and those who can be safely monitored or reassured. Integrating diverse data in ML models, including symptoms, genomic sequences, behavioral risk factors, comorbidities, laboratory test results, and other patient characteristics, may enable more accurate risk assessments compared to traditional methods [23, 24, 25, 26, 27]. Some studies have reported a 20%–25% increase in risk prediction accuracy compared to conventional methods [28, 29]. While numerous studies have focused on the application of AI in medical imaging [29, 30, 31, 32, 33, 34], there remains a notable gap in research concerning ML's capabilities for risk prediction and risk stratification in clinical practice, an area requiring further exploration to fully realize its potential in supporting early cancer diagnosis.

This systematic review aims to examine and synthesize studies on ML models development for cancer risk prediction. By specifically focusing on models that include data on signs and symptoms, in combination with other clinical and patient characteristics, the review aims to identify ML models that could support doctors' decision‐making on referrals and diagnostic work‐up in clinical practice, when patients present with potential cancer symptoms. While previous meta‐analyses and systematic reviews have largely concentrated on imaging‐based AI tools, genomic classifiers, or population‐level screening models, the present review uniquely addresses the use of ML algorithms in symptom‐based cancer risk prediction, a clinically distinct and understudied phase of the diagnostic pathway. Previous meta‐analyses have indicated that the additive value of ML over conventional statistical methods for risk prediction is uncertain [35]. This uncertainty carries important clinical implications, indicating that current ML‐based approaches should be regarded as complementary to, rather than replacements for, conventional statistical models. Robust evidence from external validation studies and head‐to‐head comparative analyses is still required to determine whether ML techniques can achieve meaningful improvements in predictive accuracy, calibration, and clinical utility beyond established methods. In this context, our review extends beyond performance comparison to provide a critical appraisal of methodological rigor, including assessments of risk of bias, validation strategies, and reporting transparency, which have often been inconsistently evaluated in prior literature. By adopting this approach, we aim to delineate the current landscape of symptom‐based ML research, highlight existing methodological and reporting gaps, and inform future studies aiming for robust, transparent, and clinically applicable AI integration in oncology.

## 2 Materials and Methods

The review has been conducted and reported following the guidelines of the Cochrane Handbook for Systematic Reviews and the Preferred Reporting Items for Systematic Reviews and Meta‐Analyses (PRISMA) statement [36].

### 2.1 Search Strategy

A systematic search was performed to identify published studies from 2014 to 2024 that developed ML algorithms for cancer risk prediction, incorporating symptoms and other relevant clinical, genetic, socio‐demographic, and lifestyle risk factors. A comprehensive literature search was conducted across four electronic databases: PubMed/MEDLINE, EMBASE, Scopus, and Web of Science. The search string used for PubMed is provided in Table S1. Search strategies for other databases were adapted based on this string to accommodate the specific syntax and indexing of each database. Additional articles were retrieved by screening references of included studies or consulting experts in the field. Papers were eligible for inclusion if they described original studies utilizing ML tools and algorithms for cancer risk prediction, which included at least one risk factor that was a clinical sign or symptom.

As the aim was to identify ML models that could support doctors' decision‐making when patients present with symptoms, we excluded papers that did not consider symptoms in their models. This exclusion criterion was applied because our review specifically aimed to identify ML models that could be applied in clinical settings for supporting decision‐making when patients present to their doctor with symptoms potentially indicative of cancer. Models developed for population‐level screening or risk stratification, not including symptoms among input variables, have a different target group and context of application, and were therefore outside the scope of this review. Our review included studies comparing ML predictive models with traditional or other ML‐based models. Reports published from January 1, 2014, to May 17, 2024, were eligible for inclusion; the final search update was conducted on May 17, 2024. All inclusion and exclusion criteria are described below.

#### 2.1.1 Inclusion Criteria

- Studies that developed ML‐based methods for predicting a primary cancer.
- Studies that used at least a clinical sign/symptom for cancer prediction in the final model.
- Studies that provided at least one quantitative performance metric for the predictive model (e.g., area under the receiver operating characteristic curve [AUC], sensitivity, specificity, calibration, etc.).
- Original studies that used observational data (including cohort studies, case–control studies, or randomized controlled trials [RCTs]).
- Studies that predicted the risk of incident cancer when patients present with symptoms, rather than studies focused on risk of cancer diagnosis in the general population, prognosis, or cancer recurrence.

#### 2.1.2 Exclusion Criteria

- Systematic reviews or conference abstracts.
- Studies that did not use ML‐based models. We recognize there is no clear split between traditional statistical methods and ML. For this review, ML was defined as a set of computational algorithms capable of automatically identifying patterns and learning from data without prespecified parametric relationships between variables. This category included supervised, unsupervised, and ensemble methods such as random forest (RF), support vector machines (SVM), k‐nearest neighbors (KNN), decision trees (DT), gradient boosting, and deep learning models (CNN, ANN, LSTM). Traditional statistical methods were defined as approaches based on explicit model specification and hypothesis‐driven relationships, including logistic regression, Cox regression, and linear models. Studies employing regularized regression (e.g., LASSO, Ridge, Elastic Net) or hybrid designs were classified based on their dominant analytical framework.
- Studies that did not include at least one clinical sign or symptom as a potential predictor in the final model.
- Non‐English language articles or studies published before 1 January 2014.

### 2.2 Data Collection and Synthesis of Results

Study selection occurred in two phases: initial screening of titles and abstracts, followed by full‐text review of potentially eligible articles. Two reviewers (S.B. and R.C.) independently assessed the studies at each stage. Disagreements were resolved through discussion, or by involving a third senior researcher if necessary (F.P.). Data were extracted using a pre‐defined data extraction form in Excel, which included clear operational definitions and examples for each predictor category. The data extraction form was piloted to ensure consistent interpretation of variables and domains across reviewers. Two reviewers independently extracted the data from all included studies. Any discrepancies were discussed and resolved through consensus, with arbitration by a senior reviewer when required. This process ensured reproducibility and standardization in the categorization of predictors across heterogeneous studies. Extracted data included key characteristics of each included study (publication year, authors, study period, country, and type of cancer), information on the development of the prediction model (ML methods used, data sources, data input, sample size, number of predictive variables). Extracted data included key characteristics from each included study (publication year, authors, study period, country, and type of cancer), information on the development of the prediction model (ML methods used, data sources, data input, sample size, number of predictive variables).

We also systematically extracted and categorized all variables used for model training as reported in each study. These were grouped into six domains: symptoms/signs, socio‐demographic characteristics, comorbidities, behavioral/lifestyle factors, laboratory and diagnostic tests, and genetic information. A detailed summary of these predictive parameters is provided in Table S2.

Predictive factors were categorized into demographics, signs and symptoms, comorbidities, behavioral/lifestyle factors, laboratory tests, and others. Key performance metrics were collected (AUC, sensitivity, specificity, VPP, VPN, *R* ^2^, D‐statistics, F1, calibration, precision). Data extraction was performed in duplicate, and discrepancies were resolved by discussion. The TRIPOD adherence assessment form was used to evaluate the adherence rate for the reporting of individual studies [37].

### 2.3 Risk of Bias

The methodological quality of the studies was independently assessed by two reviewers (F.P., A.C.D.A.) using the QUADAS‐AI tool. QUADAS‐AI is specifically designed for evaluating AI‐centric diagnostic precision and accuracy studies, addressing unique terminology and criteria relevant to AI applications. This tool extends the QUADAS‐2 framework to account for methodological aspects specific to AI in diagnostic research, including data preprocessing, model training, and validation. As the included studies primarily evaluated ML algorithms for diagnostic risk prediction based on clinical symptoms, QUADAS‐AI was deemed more appropriate. Articles and their supplementary materials were meticulously screened to assess potential biases and concerns regarding applicability.

## 3 Results

### 3.1 Study Selection and Study Characteristics

The database search identified a total of 5646 studies, among which we selected 4197 unique studies after removing duplicates. Following title and abstract screening, we excluded 4121 papers, as they did not consider signs or symptoms, leaving 76 papers for further assessment. We included a total of 34 studies on ML models' development and validation for early cancer diagnosis and risk stratification.

**FIGURE 1:**
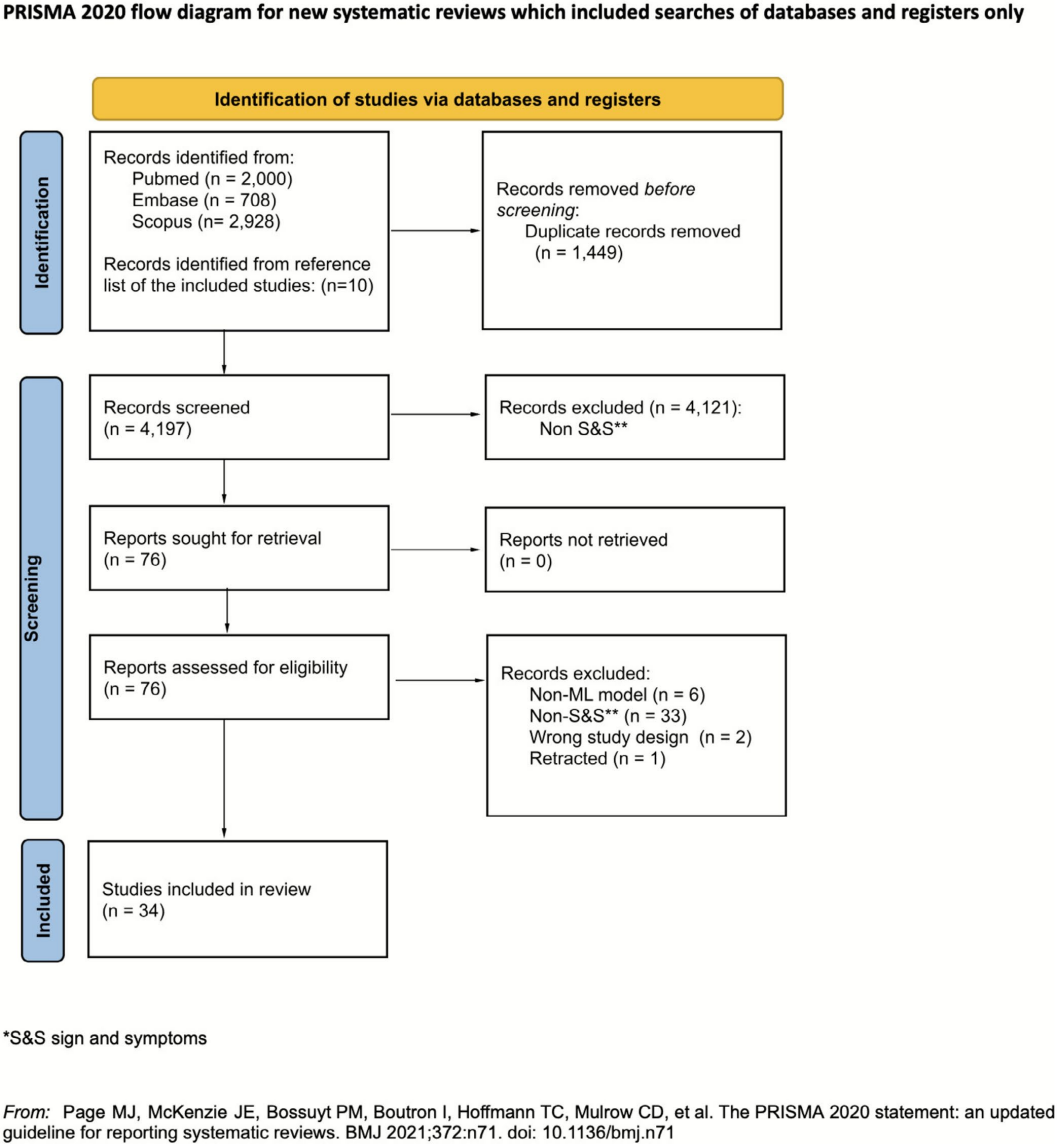
PRISMA flow diagram of study search, screening, assessment, and extraction.

A PRISMA flowchart diagram illustrating the article selection process is presented in Figure 1.

**FIGURE 2:**
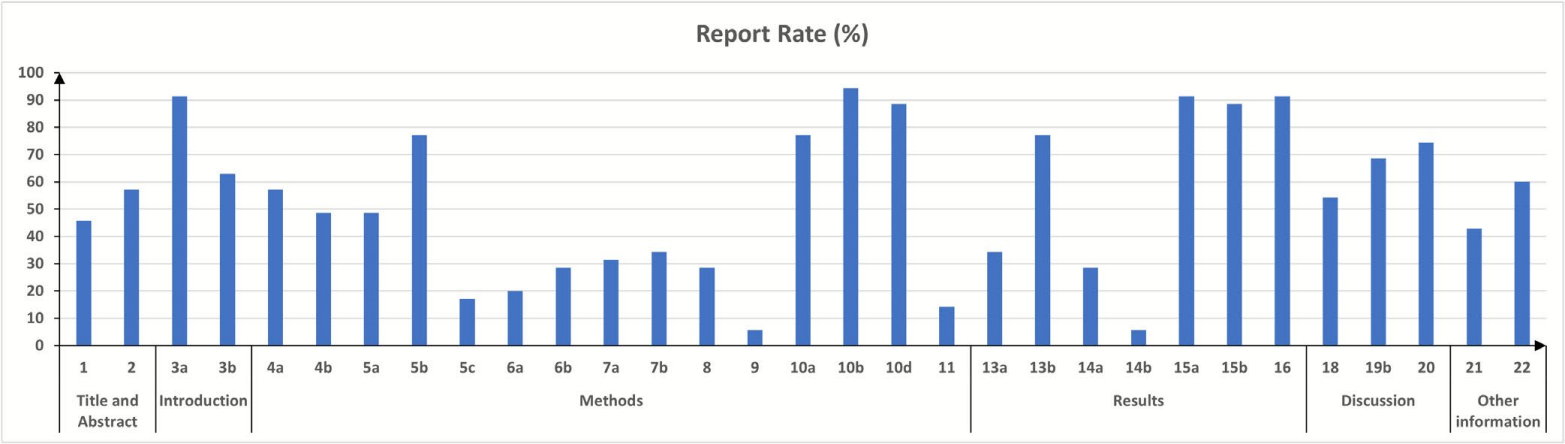
Adherence to Transparent Reporting of a Multivariable Prediction Model for Individual Prognosis or Diagnosis (TRIPOD) checklist per reporting item.

While we searched studies published between 2014 and 2024, the majority were published in the past five years (*n* = 25) [38, 39, 40, 41, 42, 43, 44, 45, 46, 47, 48, 49, 50, 51, 52, 53, 54, 55, 56, 57, 58, 59, 60, 61, 62]. Included studies were from three geographic areas (based on the data origin): Europe (*n* = 7) [40, 52, 56, 57, 61, 63, 64], North America (*n* = 7) [38, 44, 46, 50, 55, 65, 66], and Asia (*n* = 17) [39, 41, 42, 43, 45, 47, 48, 49, 51, 53, 59, 60, 62, 67, 68, 69, 70] (Table 1). Most of them (*n* = 32) [39, 40, 41, 42, 43, 44, 45, 46, 47, 48, 49, 50, 51, 52, 53, 54, 55, 56, 57, 58, 59, 60, 61, 62, 63, 64, 65, 66, 68, 69, 70, 71] focused on developing and validating ML models for cancer risk prediction by assessing performance metrics such as accuracy, sensitivity, and AUC. Among the included studies, two [38, 58] employed multiple AI models and 27 [39, 40, 41, 42, 44, 45, 46, 47, 48, 49, 50, 51, 52, 53, 54, 55, 56, 57, 58, 59, 60, 61, 62, 65, 66, 68, 70] reported more than one performance metric. The median (IQR [range]) overall adherence rate to TRIPOD checklist items was 55 (40–68 [10–81]) % (Table 1), with a breakdown of reporting per individual item shown in Figure 2.

**TABLE 1:** Study details including year, authors, study period, country of publication, and cancer site.

Year	Authors	Study period	Setting	Cancer type	ML methods	Objective	TRIPOD adherence rate (%)
2021	Alam et al. [38]	NA	USA	Mesothelioma	Apriori algorithm	Propose a ML approach for Identification of malignant mesothelioma etiological factors in an imbalanced dataset	0.32
2020	Al‐Juboori et al. [39]	NA	Iraq	Breast Cancer	ANN LDA RF	Suggest a classifiers in accordance with super symptoms analysis and predict the class in the dataset by using different algorithms	0.42
2019	Bhuta et al. [67]	NA	India	Brain Cancer	DT NB	Propose an algorithm (DT and NB) that predicts the type of brain tumor with accuracy	0.10
2022	Briggs et al. [40]	2013	UK	Oesophago‐gastric cancer	LR NB RF SVM XGBoost	Assess suitability of an ML‐based approach against the UK Cancer ogRAT for risk prediction of oesophago‐gastric cancer, using data derived from primary care EHRs	0.74
2023	Chen et al. [41]	January 2018–June 2021	China	Nasopharyngeal Carcinoma	XGBoost	Build practical ML models from EHR data for nasopharyngeal carcinoma risk prediction in different clinical settings	0.58
2021	Chen et al. [43]	NA	China	Breast Cancer	NLP	Propose an online textual symptomatic assessment chatbot based on question‐and‐answer (Q&A) approach weighted scoring for female breast cancer prescreening	0.55
2024	Chen et al. [42]	2001–2008	China	Nasopharyngeal Carcinoma	LGBM LR MARS RF XGBoost	Validate a ML model utilizing symptom‐related diagnoses and procedures in medical records to predict nasopharyngeal carcinoma occurrence and reduce the prediagnostic period	0.81
2021	Chen et al. [44]	2008–2017	USA	Pancreatic Cancer	XGBoost	Evaluate a ML approach to help identify patients with early‐stage pancreatic cancer from clinical data within EHR	0.68
2019	Chicco et al. [68]	2011	Turkey	Mesothelioma	DT OR PNN PBNN RF	Use ML for prediction of diagnosis and feature selection on mesothelioma patient health records	0.58
2021	Choudhury et al. [46]	NA	USA	Mesothelioma	AdaBoost CC KLR MLP SGD s‐Pegasos VFDT VP	Use ML recommending the best fit model for early diagnosis and prognosis for mesothelioma	0.45
2023	Dirik et al. [45]	2013	Turkey	Lung cancer	DT Gboost FLM LR NB RF SVM	Develop an automated model that can detect early‐stage lung cancer based on nine ML learning	0.42
2020	Duan et al. [47]	NA	China	Lung cancer	ANN DT SVM	Propose a three‐layer diagnosis system (C5.0, ANN, SVM) for lung cancer based on multidimensional variables	0.61
2023	Erdemoglu et al. [48]	January 2015–May 2022	Turkey	Endometrial cancer	CATboost LR MP NB RF XGBoost	Analyze different artificial intelligence methods to help in clinical decision‐making and the prediction of endometrial intraepithelial neoplasia and endometrial cancer risks in pre‐ and postmenopausal women	0.68
2014	Goryński et al. [63]	2006	Poland	Lung cancer	BFGS GD MLP	ANN model can be useful for the selection of the group with highest risk of developing lung cancer	0.55
2022	Hossain et al. [49]	NA	Bangladesh	Leukemia	Adaboost ANN DT k‐Nearest Neighbor LR NB RF	Propose a ML model that predicts the likelihood of early‐stage leukemia based on symptoms only	0.52
2018	Hu et al. [65]	NA	USA	Mesothelioma	BPSSAE ELM GA RF	Develop a deep learning method to automatically diagnose mesothelioma	0.45
2017	Kinar et al. [69]	2008	Israel	Colorectal cancer	DT GBM MeScore	Perform analysis of a ML flagging system used to identify a group of individuals at a high risk for colorectal cancer	0.55
2022	Lindvall et al. [50]	(1) Eletronic Health Records notes from DFCI—January 2016 and December 2019 (2) EHR notes from MIMIC‐III—January 2008 and December 2012	USA	Various cancer (breast, GI, thoracic, gynecologic)	BERT‐base ClinicalBERT ClinicalELECTRA‐small ClinicalXLNet DistilBERT‐base ELECTRA‐small Longformer‐base RoBERTa‐base XLMRobERTa‐base XLNet‐bas	Develop and test a deep learning model for symptom extraction from unstructured clinical notes and externally validate the method in a data set from another health care system	0.65
2021	Lo et al. [51]	January 2005–December 2013	China	Bladder cancer	DT LR MLP RF SVM	Propose a procedure integrating ML and analytic hierarchy process to predict delayed diagnosis of bladder patients with hematuria	0.71
2021	Malhotra et al. [52]	January 2005–June 2009	UK	Pancreatic cancer	LR RF	Identify a sub‐population of patients at high risk of diagnosis for pancreatic cancer using ML techniques applied to primary care data	0.71
2023	Masadah et al. [53]	January 2023–June 2023	Indonesia	Endometrial cancer	ANN DT	Propose ML model using demographic factors, gynecological symptoms and β‐catenin for endometrial hyperplasia and carcinoma	0.81
2022	Mezher et al. [54]	NA	Saudi Arabia, Italy	Lung cancer	GFS SVM	Use SVM to improve the classification accuracy of genetic folding in classifying lung cancer into malignant and benign	0.29
2021	Mishra et al. [55]	NA	USA	Lung cancer	heuristic‐based GBFS‐Random forest model	Propose a sustainable IoHT based computationally intelligent healthcare monitoring system for lung cancer risk detection	0.39
2019	Muhammad et al. [66]	1997 to 2017 (dataset NHIS) November 1993 to July 2001 (PLCO)	USA	Pancreatic cancer	ANN	Develop an ANN to calculate risk for pancreatic cancer in the general population and to identify a high‐risk population in a cost‐effective manner by utilizing easily available personal health data	0.65
2018	Mukherjee et al. [70]	NA	Turkey	Mesothelioma	MLPE, SVM	Try to find the important input variables for mesothelioma disease diagnosis using strong DM techniques	0.35
2022	Nemlander et al. [56]	September 2014–November 2015	Sweden	Lung cancer	SGB	Predict lung cancer using ML on data from a symptom e‐questionnaire for smokers	0.77
2023	Nemlander et al. [57]	2011	Sweden	Colorectal cancer	SGB	Develop a predictive model for identifying non‐metastatic CRC among patients using diagnostic data	0.74
2021	Oliver et al. [58]	NA	India, Egypt, USA, and UK	Lung cancer	Hybrid ensemble algorithm	Detect of lung carcinoma using ML by identifying it at an earlier stage based on the existing symptoms	0.26
2019	Rajan et al. [71]	NA	Various country	Lung cancer	MNN	Propose a model for applying data mining techniques to detection of lung cancer at the very early stage	0.23
2020	Senturk et al. [59]	NA	Turkey	Mesothelioma	ANN GBT kNN RF SVM	Propose a ML based early diagnosis system for mesothelioma disease	0.42
2024	Wani et al. [60]	NA	India	Lung cancer	CNN XGBoost	Focus on employing a deep‐learning algorithm to detect lung cancer, and use XAI methods, notably SHAP	0.39
2020	Weegar et al. [61]	2007–2014	Sweden	Cervical cancer	BNB CNB RF SVM	Use ML for predicting cervical cancer from EHR	0.58
2016	Xie et al. [64]	1995–1997	Sweden	Oesophago‐gastric cancer	LR RF	Develop a prediction model for valid estimation of the absolute 5‐year risk of esophageal adenocarcinoma	0.77
2022	Zadsafar et al. [62]	NA	Turkey	Mesothelioma	BHHO+DT HHO	Propose a model (BHHO + DT) to diagnose mesothelioma using patient health record	0.55

Abbreviations: ANN, Deep Learning Artificial Neural Network; AUC, Area under curve; BHHO, Binary Harris Hawk optimization; BNB, Bernoulli Naive Bayes; BP, Backpropagation Algorithm; CC, Clojure Classifier; CNB, Complement Naive Bayes; DS, Dataset; DT, Decision Tree; EHR, Electronic health record; ELM, Extreme Learning Machine; GA, Genetic algorithm; GBFS, Gradient Boosted Feature Selection‐Random forest model; GBT, Gradient booster trees; GFS, Genetic Folding Strategy; KLR, Kernel Logistic Regression; k‐NN, k‐Nearest Neighbor; LDA, Linear Discriminant Analysis; LR, Logistic Regression; MLP, Multilayer Perceptron; MLPE, Multilayer Perceptron Ensemble; NB, Naive Bayes; PBNN, Perceptron‐based neural network; PNN, Probabilistic neural network; RF, Random Forest; SGD, Stochastic gradient descent; s‐Pegasos, Primal Estimated Sub‐gradient Solver for Support Vector Machine; SSAE, Stacked Sparse Autoencode; SVM, Support Vector Machine; VFDT, Hoeffding Tree; VP, Voted perceptron; XGBoost, Extreme Gradient Boosted Decision Tree.

**FIGURE 3:**
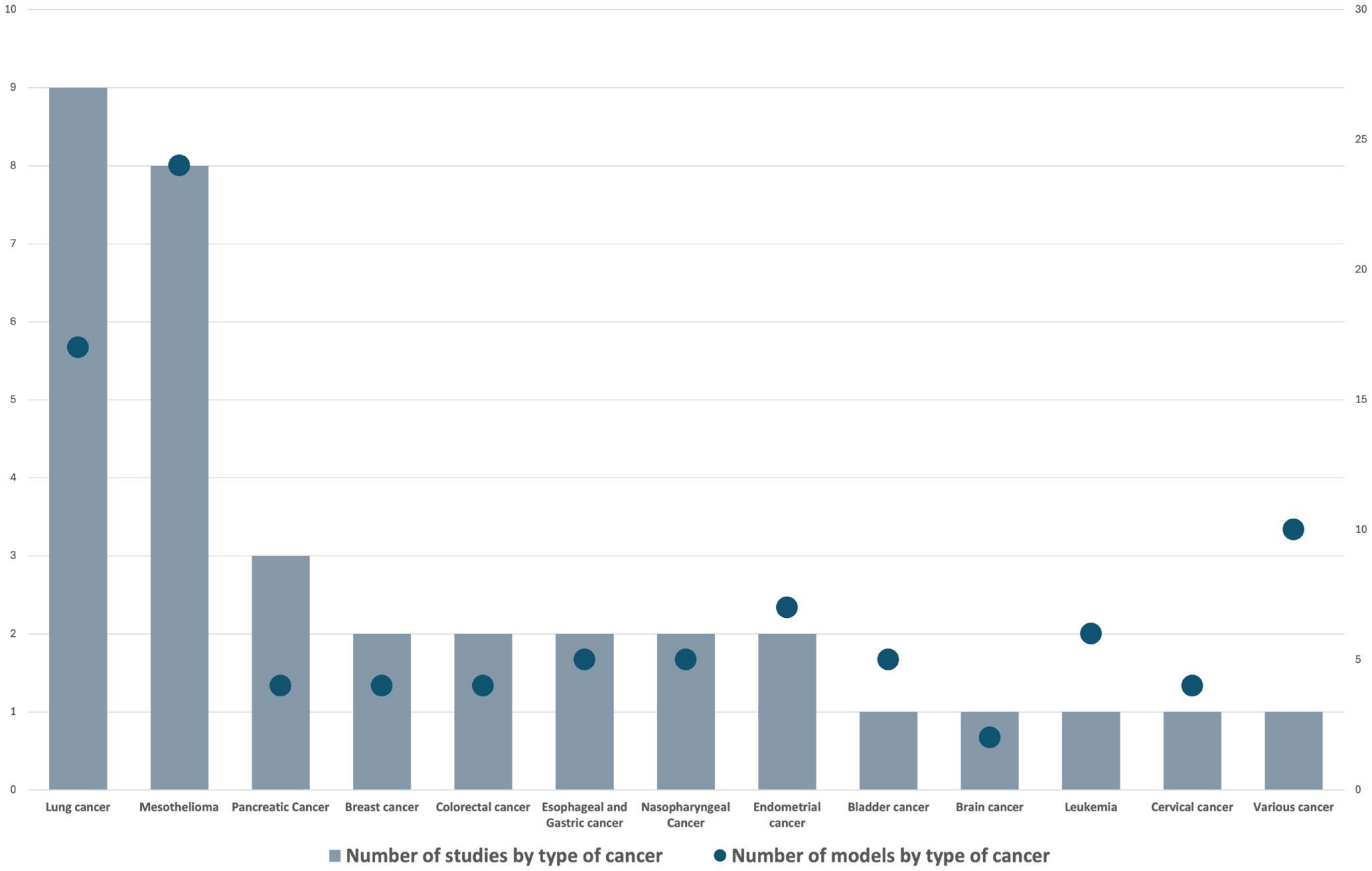
Number of studies examining different cancer types and employing different ML models.

We identified most studies using ML techniques to predict risk of cancer diagnosis in symptomatic patients for lung cancer (*n* = 9 studies) [45, 47, 54, 55, 56, 58, 60, 63, 71], mesothelioma (*n* = 7) [38, 46, 59, 62, 65, 68, 70], gastrointestinal cancer (*n* = 4) [40, 57, 64, 69] and pancreatic cancer (*n* = 3) [44, 52, 66] (Figure 3).

The Random Forest (RF) algorithm was the most commonly employed technique (*n* = 12) [39, 40, 42, 45, 48, 49, 51, 52, 59, 61, 64, 68], followed by Support Vector Machines (SVM) (*n* = 8) [40, 45, 47, 51, 54, 59, 61, 70], Decision Trees (DT) (*n* = 8) [45, 47, 49, 51, 53, 67, 68, 69], Logistic Regression (LR) (*n* = 8) [40, 42, 45, 48, 49, 51, 52, 64], and XGBoost (*n* = 6) [40, 41, 42, 44, 45, 48] (Table 2). Nine studies utilized deep learning models [39, 47, 49, 50, 53, 59, 65, 66, 71]. Additionally, five hybrid models (models incorporating both ML and more traditional biostatistical methods) were included [55, 58, 60, 62, 70].

**TABLE 2:** ML models for cancer risk prediction in symptomatic patients employed in the reviewed studies.

ML models	Number of studies	Number of variables (range)
**Machine learning**		
Random Forest (RF)	12	3–75,000^a^
Support Vector Machine (SVM)	8	14–75,000^a^
Decision Tree (DT)	8	5–33
Logistic Regression (LR)	8	3–50
Extreme Gradient Boosted Decision Tree (XGBoost)	7	3–582
Naive Bayes (NB)	5	3–18
Multilayer perceptron (MLP)	4	3–47
AdaBoost	2	16–35
k‐Nearest Neighbor (k‐NN)	2	16–34
Stochastic Gradient Boosting (SGB)	2	10–184
Multivariate Adaptive Regression Splines (MARS)	1	14
Linear Discriminant Analysis (LDA)	1	8
One Rule	1	33
Clojure Classifier (CC)	1	35
Kernel Logistic Regression (KLR)	1	35
Primal Estimated Sub‐gradient Solver for Support Vector Machine (s‐Pegasos)	1	35
Complement Naive Bayes (CNB)	1	75,000^a^
Bernoulli Naive Bayes (BNB)	1	75,000^a^
Hoeffding Tree (VFDT)	1	35
Association Rule Mining (Apriori algorithm)	1	26
Light Gradient Boosting Machine (LGBM)	1	14
Probabilistic neural network (PNN)	1	33
Perceptron‐based neural network (PBNN)	1	33
Voted perceptron (VP)	1	35
Stochastic gradient descent (SGD)	1	35
Catboost	1	3
Gradient descent (GD)	1	47
BFGS (Broyden‐Fletcher‐Goldfarb‐Shanno)	1	47
ReliefF algorithm	1	19
Genetic algorithm (GA)	1	19
Extreme Learning Machine (ELM)	1	19
MeScore algorithm	1	9
Gradient boosting machines (GBM)	1	9
Genetic Folding Strategy (GFS)	1	NA
Gradient booster trees (GBT)	1	34
Natural language processing (NLP)	1	16
**Deep learning**		
Artificial Neural Network (ANN)	6	5–34
Multi‐Class Neural Networks (MNN)	1	NA
Backpropagation (BP) Algorithm	1	19
Stacked Sparse Autoencoder (SSAE)	1	19
BERT‐base	1	80
ClinicalBERT	1	80
XLNet‐base	1	80
ClinicalXLNet	1	80
DistilBERT‐base	1	80
RoBERTa‐base	1	80
XLMRobERTa‐base	1	80
ELECTRA‐small	1	80
ClinicalELECTRA‐small	1	80
Longformer‐base	1	80
**Combined models**		
Multilayer Perceptron Ensemble (MLPE)	1	34
Hybrid ensemble	1	15
ConvXGB	1	16
GBFS‐Random forest model	1	16
BHHO+DT Binary Harris Hawk optimization (HHO)	1	17

^a^ Study [61] applied lemmatization to reduce variability by grouping different word forms but still identified around 75,000 distinct events. This was partly due to variations in how similar events were phrased, such as “fractured patella” and “patella fracture,” which were treated as separate events.

### 3.2 Type of Data Included in Risk Prediction Models

The studies were based on either non‐publicly available datasets, with access restricted to researchers belonging to specific institutions (*n* = 23) [38, 39, 41, 42, 43, 46, 47, 48, 49, 51, 53, 56, 57, 59, 61, 62, 63, 64, 65, 67, 68, 69, 70], or datasets available for the wider research community, with access typically requiring a research application and ethics approval (*n* = 10) [40, 44, 50, 52, 54, 55, 58, 60, 66, 71]. Four studies [49, 50, 57, 66] utilized two different datasets. Of these, two [49, 50] reported using one dataset for training and the other for testing.

Restricted access datasets originate from hospitals or primary care settings, with nine studies [39, 43, 47, 48, 49, 56, 61, 63, 64] using hospital data, one [57] using primary care data, and one study [52] including both primary and secondary care data. Three studies [45, 60, 66] derived their data from surveys. Datasets available for the wider research community, typically provided by government agencies or research institutions, were employed in 10 studies [40, 44, 50, 52, 54, 55, 58, 60, 66, 71] and are summarized in Table 3. Only one study [50] included clinical free text notes.

**TABLE 3:** Datasets available to the wider research community for ML‐based cancer risk prediction for symptomatic patients based on included studies.

Dataset name	Description	Population	Data link	Studies	Ethical approval required
UCI Machine Learning repository	The UCI Machine Learning Repository is a collection of databases, domain theories, and data generators that are used by the machine learning community for the empirical analysis of ML algorithms. It contains 665 datasets regarding various conditions, cancer, infection and health problem.	World	http://www.archive.ics.uci.edu/datasets	Mishra et al.	No
(UK) Clinical Practice Research Datalink (CPRD), previously known as General Practice Research Database	The General Practice Research Database (GPRD), now called Clinical Practice Research Datalink (CPRD), is the world's largest database of anonymised longitudinal clinical records from primary care. The database has an international reputation for health research in various fields. The CPRD includes around 5 million patients from around 590 primary care practices throughout the UK.	UK	https://digital.nhs.uk/data‐and‐information/data‐collections‐and‐data‐sets/data‐collections/general‐practice‐data‐for‐planning‐and‐research	Briggs et al., Malhotra et al.	Yes
Optum electronic medical records dataset	Optum consolidates and enriches electronic health record (EHR) data that span therapeutic areas and sites of care. Optum remains at the forefront of using natural language processing (NLP) to provide meaning, structure and context to clinical notes.	USA	https://www.optum.com/en/business/life‐sciences/real‐world‐data/ehr‐data.html	Chen et al.	Yes
Medical Information Mart for Intensive Care III (MIMIC‐III) dataset	MIMIC‐III (Medical Information Mart for Intensive Care III) is a large, freely‐available database comprising de‐identified health‐related data associated with over forty thousand critical care patients. The dataset consists of 112,000 clinical reports records and 1159 top‐level ICD‐9 codes. The database includes demographics, vital signs, laboratory test results, procedures, medications, caregiver notes, imaging reports, and mortality (both in and out of hospital).	USA	https://mimic.mit.edu/	Lindvall et al.	Yes
Lung cancer dataset from Kaggle	Kaggle is a platform for data science and machine learning that provides users with opportunities to work on various datasets, participate in competitions, and collaborate with other data scientists.	World	https://www.kaggle.com/	Mezher et al.	No
National Health Interview Survey (NHIS) dataset	NHIS uses an address‐based, complex clustered sample of housing units, yielding data representative of the civilian noninstitutionalized US population. NHIS provides data on a broad range of health topics collected through personal household interviews. Survey results have been instrumental in providing data to track health status, health care access, and progress toward achieving national health objectives.	USA	https://www.cdc.gov/nchs/nhis/index.htm	Muhammad et al.	No
The Prostate, Lung, Colorectal, and Ovarian (PLCO) dataset	The Prostate, Lung, Colorectal, and Ovarian (PLCO) Cancer Screening Trial is a randomized, controlled trial, conducted by the National Cancer Institute (NCI) to determine whether certain screening exams reduce mortality from prostate, lung, colorectal and ovarian cancer. Approximately 155,000 participants were enrolled between November 1993 and July 2001. The primary endpoint within the PLCO trial was cause‐specific mortality through 13 years of follow‐up. Secondary endpoints included screening compliance and positivity rates, diagnostic follow‐up, sensitivity and specificity, cancer incidence and stage distribution, and all‐cause mortality.	USA	https://cdas.cancer.gov/plco/	Muhammad et al.	Yes
Data.world repository	Data.world offers a variety of datasets on different topics that can be used for analysis, research, and projects.	World	https://data.world/	Oliver et al., Rajan et al., Wani et al.	No

Most studies used single data sources. However, two studies [40, 69] combined different types of data to create multimodal predictive models, integrating electronic health records with cancer markers or medical imaging data.

There was considerable variability in the sample sizes used to develop cancer risk prediction models. The cancer sample sizes (cases) ranged from a minimum of 17 patients [53] to 7471 patients [40]. The maximum total sample size, including both cases and controls, was 964,579 patients. Additionally, two studies [41, 50] included an external validation sample, bringing the total to over one million patients. Two studies [70, 71] did not specify the number of cases and controls, six studies [39, 43, 47, 48, 50, 55] lacked a control sample, and one study [60] provided only the total sample size without distinguishing between cases and controls. Regarding the sample size, three studies [41, 48, 68] divided the sample into training, testing, and validation sets, with six studies [40, 44, 59, 62, 65, 66] providing the percentages of the sample used for training (ranging from 70% to 80%) and testing (ranging from 20% to 30%) (Table S2).

Only 11 studies [39, 41, 44, 49, 51, 53, 54, 58, 61, 65, 66] reported their methods for handling missing data before training. The most frequent approach, employed by four studies [51, 54, 58, 61], was complete case analysis. Five studies [39, 49, 51, 54, 58] removed missing data as a part of pre‐processing and data cleaning (complete case); two studies [53, 65] reported no missing data, one [44] used statistical modeling to address missing data in real‐time systems, another [66] employed one‐hot encoding with missing values set to −1, and one study [41] relied on XGBoost handling missing data effectively.

### 3.3 Risk Predictors

The initial number of variables considered for risk prediction ranged from 15 to 18,220, with the final selection ranging from 3 [48] to 582 [44] (excluding one article [61] with 75,000 variables). Table S3 summarizes the risk predictors across the included studies. Most articles (*n* = 33) included cancer‐specific symptoms or signs. The six lung cancer studies [45, 47, 55, 60, 63, 71] considered both specific symptoms/signs (cough, chest pain, dyspnea, wheezing, yellow fingers) and generic symptoms (fever, fatigue, appetite loss), while three studies [54, 57, 58] used only specific symptoms. Of the three pancreatic cancer articles, two reported specific (pruritus) and generic symptoms (pain, weight loss, fatigue, anorexia, anxiety, weakness, fever) [44, 66], while the third listed only one comorbidity (asthma) [52]. Further details are in Tables S3 and S4.

Twenty‐seven studies described a model including sociodemographic characteristics, with age (*n* = 27) [38, 39, 40, 42, 44, 45, 46, 47, 48, 50, 51, 53, 54, 55, 56, 58, 59, 60, 61, 62, 63, 64, 65, 66, 68, 69, 70], gender (*n* = 21) [38, 42, 43, 44, 45, 46, 47, 50, 51, 54, 56, 58, 59, 62, 63, 64, 65, 66, 68, 69, 70], geographic area (*n* = 9) [38, 46, 51, 59, 62, 63, 65, 68, 70], and education level (*n* = 4) [44, 56, 63, 64] being the most common.

Seventeen articles [41, 43, 44, 45, 48, 52, 53, 54, 55, 57, 58, 60, 61, 63, 64, 66, 69] reported comorbidities, either cancer‐specific (e.g., colorectal cancer: hemorrhoids) or general chronic conditions (e.g., hypertension, allergy, diabetes). Eight articles [43, 47, 48, 49, 52, 55, 63, 66] incorporated genetic data. Lifestyle factors such as smoking (*n* = 20) [38, 45, 46, 47, 48, 49, 52, 54, 55, 56, 58, 59, 60, 62, 63, 64, 65, 66, 68, 70] and alcohol drinking (*n* = 8) [45, 47, 52, 54, 55, 58, 66, 69] were also included. For mesothelioma, studies (*n* = 7) [38, 46, 59, 62, 65, 68, 70] considered asbestos, C‐reactive protein, and pleural protein exposure as a key risk factor. In contrast, gastrointestinal cancer studies [40, 57, 64, 69] identified predictive factors such as diabetes, age, and hyperlipidemia. Most studies focused on the predictive nature of associations without inferring causality.

#### 3.3.1 Grouping Studies by Data Input Type

To address the substantial heterogeneity in model inputs, we categorized the studies based on the types of data included in the final ML models. Six categories were defined: (1) symptoms only; (2) symptoms + sociodemographic variables (e.g., age, sex); (3) symptoms + comorbidities (e.g., diabetes, hypertension); (4) symptoms + lifestyle factors (e.g., smoking, alcohol use); (5) symptoms + laboratory, genetic, or imaging data; and (6) multimodal models (including ≥ 3 of the above domains). Most studies (*n* = 27) included sociodemographic data; 17 studies [41, 43, 44, 45, 48, 52, 53, 54, 55, 57, 58, 60, 61, 63, 64, 66, 69] included comorbidities, and 20 [38, 45, 46, 47, 48, 49, 52, 54, 55, 56, 58, 59, 60, 62, 63, 64, 65, 66, 68, 70] incorporated lifestyle variables. Only a minority used genetic or laboratory data (*n* = 8) [43, 47, 48, 49, 52, 55, 63, 66], though these were more frequent in recent studies. Multimodal models, which integrated three or more data types, were observed in 8 studies [42, 44, 52, 56, 59, 60, 62, 66]. A summary classification of each study by data type is presented in Table S5.

### 3.4 Evaluation and Performance Metrics

Nearly all studies (*n* = 24) [39, 40, 46, 47, 48, 50, 51, 52, 53, 54, 56, 57, 59, 60, 61, 62, 63, 64, 66, 67, 68, 69, 70, 71] used internal validation, which reduced the available sample size. Cross‐validation techniques were applied in three studies [39, 50, 53], while K‐fold cross‐validation was the most used method (*n* = 16) [40, 44, 46, 48, 49, 51, 54, 56, 57, 58, 60, 61, 64, 66, 68, 70]. Only two studies [42, 50] conducted external validation to assess their predictive model's generalizability. Of these, one study [50] was externally validated using 100 physician notes randomly selected from a dataset. The other article [42] used a separate dataset of 1 million individuals resident in Taiwan.

The evaluation metrics used in the reviewed studies varied widely. Most studies (*n* = 33) reported performance metrics, most commonly the AUC (*n* = 22) [40, 41, 42, 44, 45, 46, 47, 48, 49, 51, 52, 53, 54, 56, 57, 58, 61, 63, 64, 65, 66, 70], accuracy (*n* = 23) [39, 40, 41, 42, 43, 45, 46, 47, 48, 49, 51, 53, 54, 55, 56, 58, 59, 60, 62, 65, 68, 70, 71], specificity (*n* = 15) [41, 42, 44, 45, 47, 51, 52, 53, 55, 56, 57, 61, 65, 66, 68], precision (*n* = 8) [40, 41, 45, 49, 50, 58, 61, 62], sensitivity (*n* = 24) [39, 40, 41, 42, 44, 45, 47, 48, 49, 50, 51, 52, 53, 54, 55, 56, 57, 58, 59, 60, 61, 65, 66, 68], and F1 score (*n* = 11) [40, 45, 48, 50, 55, 58, 60, 62, 65, 68, 70]. Despite the importance of calibrating ML models for predictive performance assessment, only two studies (*n* = 2) [40, 43] evaluated model calibration (Table 4).

**TABLE 4:** Performance measures of ML‐based predictive models.

Authors	*R* ^2^ (%)	Sensitivity (%)	Specificity (%)	Accuracy (%)	Precision	F1	C‐statistics (AUC)	PPV	NPV	Calibration	Development, testing, internal/external validation
Alam et al.	NA	NA	NA	NA	NA	NA	NA	NA	NA	NA	NA
Al‐Juboori et al.	NA	ANN = 72.7%; LDA = 64.2%; RF = 72.2%	NA	ANN = 72.7%; LDA = 64.2%; RF = 68.4%	NA	NA	NA	NA	NA	NA	Training and Testing
Bhuta et al.	NA	NA	NA	NA	NA	NA	NA	NA	NA	NA	NA
Briggs et al.	NA	SVM = 53%; LR = 58%; XGBoost = 54%	NA	SVM = 89%; LR = 89%; XGBoost = 89%	SVM = 90%; LR = 90%; XGBoost = 91%	SVM = 65%; LR = 68%; XGBoost = 66%	SVM = 87%; LR = 87%; XGBoost = 87%	NA	NA	Observed to predicted risk	Testing Internal validation—Cross‐validation folds
Chen et al.	NA	79% (95% CI 76–80)	96% (95% CI 95–96)	88% (95% CI 87–89)	94% (95% CI 93–95)	NA	87% (95% CI 86–88)	NA	NA	NA	Development
Chen et al.	NA	NA	NA	95.4%	NA	NA	NA	NA	NA	NA	Training
Chen et al.	NA	Development set = 64% Training set = 81% Validation set = 79%	Development set = 81% Training set = 81% Validation set = 80%	Development set = 72% Training set = 81% Validation set = 79%	NA	NA	Development set = 83% Training set = 90% Validation set = 89%	NA	NA	Observed to predicted risk	Development Training External validation
Chen et al.	NA	90% 80% 70% 60% 50% 40% 30% 20%	50.9% 68.2% 81.0% 89.8% 95.0% 98.5% 99.7% 99.9%	NA	NA	NA	84% (95% CI 83–85)	0.02%–0.07% 0.03%–0.10% 0.04%–0.14% 0.07%–0.23% 0.12%–0.39% 0.31%–1.03% 1.28%–4.16% 2.55%–8.03%	NA	NA	Testing Internal validation—Cross‐validation folds
Chicco et al.	NA	RF = 75%; DT = 72%; PBNN = 95%; One rule = 47%; PNN = 50%	RF = 86%; DT = 82%; PBNN = 20%; One rule = 67%; PNN = 58%	RF = 82%; DT = 79%; PBNN = 62%; One rule = 57%; PNN = 53%	NA	RF = 80%; DT = 77%; PBNN = 71%; One rule = 55%; PNN = 50%	NA	NA	NA	NA	Testing Internal validation—Cross‐validation folds
Choudhury et al.	NA	NA	NA	SGD = 100% (phase 1), 69.2% (phase 2); AdabostM1 = 100% (phase 1), 71.3% (phase 2); KLR = 100% (phase 1), 69.5% (phase 2); MLP = 100% (phase 1), 64.1% (phase 2); VP = 70.4% (phase 1, e phase 2); VFDT = 100% (phase 1), 70.4% (phase 2); CC = 70.4% (phase 1), 70.4% (phase 2); s‐Pegasos = 99.4% (phase 1), 67.3% (phase 2)	NA	NA	SGD = 100% (phase 1), 58% (phase 2); AdabostM1 = phase 1 (100%), phase 2 (61%); KLR = phase 1 (100%), phase 2 (65%); MLP = phase 1 (100%), phase 2 (65%); VP = phase 1 (50%), phase 2 (50%); VFDT = phase 1 (100%), phase 2 (50%); CC = phase 1–2 (50%); s‐Pegasos = phase 1 (99%), phase 2 (58%)	NA	NA	NA	Testing Internal validation—Cross‐validation folds
Dirik et al.	NA	NB = 97%; LR = 95%; DT = 100%; RF = 95%; GB = 97%; SVM = 94%	LR = 45%	NB = 91%; LR = 89%; DT = 88%; RF = 90%; GB = 88%; SVM = 91%	NB = 92%; LR = 92%; DT = 88%; RF = 93%; GB = 89%; SVM = 96%	NB = 95%; LR = 93%; DT = 93%; RF = 94%; GB = 93%; SVM = 95%	NB = 67%; LR = 82%; DT = 70%; RF = 75%; GB = 71%; SVM = 44%	NA	NA	NA	Development
Duan et al.	NA	DT (C5.0.1) = 61.8%; DT (C5.0.2) = 78.1%; DT (C5.0.3) = 94.1%; ANN‐1 = 81.6%; ANN‐2 = 84.4%; ANN‐3 = 88.2%; SVM‐1 = 59.2%; SVM‐2 = 78.1%; SVM‐3 = 82.4%	DT (C5.0.1) = 73.4%; DT (C5.0.2) = 82.6%; DT (C5.0.3) = 87.5%; ANN‐1 = 65.6%; ANN‐2 = 93.4%; ANN‐3 = 93.8%; SVM‐1 = 68.8%; SVM‐2 = 87%; SVM‐3 = 87.5%	DT (C5.0.1) = 69.1%; DT (C5.0.2) = 80.7%; DT (C5.0.3) = 90.9%; ANN‐1 = 71.6%; ANN‐2 = 89.7%; ANN‐3 = 90.9%; SVM‐1 = 65.2%; SVM‐2 = 83.3%; SVM‐3 = 84.8%	NA	NA	DT (C5.0.1) = 68% (95% CI 61–74); DT (C5.0.2) = 80% (95% CI 70–89); DT (C5.0.3) = 91% (95% CI 75–98); ANN‐1 = 74% (95% CI 67–79); ANN‐2 = 89% (95% CI 80–95); ANN‐3 = 91% (95% CI 76–98); SVM‐1 = 64% (95% CI 57–71); SVM‐2 = 83% (95% CI 73–90); SVM‐3 = 85% (95% CI 68–95)	DT (C5.0.1) = 58.0%; ANN.1 = 58.5%; SVM.1 = 52.9%; DT (C5.0.2) = 75.8%; ANN.2 = 90.0%; SVM.2 = 80.7%; DT (C5.0.3) = 88.9%; ANN.3 = 86.4%; SVM.3 = 82.4%	DT (C5.0.1) = 76.4%; ANN.1 = 85.7%; SVM.1 = 74%; DT (C5.0.2) = 84.4; ANN.2 = 89.6%; SVM.2 = 85.1%; DT (C5.0.3) = 93.3%; ANN.3 = 93.8%; SVM.3 = 87.5%	NA	Development
Erdemoglu et al.	NA	50%	NA	94%	NA	59%	94%	NA	NA	NA	Testing Internal validation—Cross‐validation folds
Goryński et al.	NA	NA	NA	NA	NA	NA	Learning set = 100% Testing set = 99% Validating set = 100% Learning, testing and validating test = 100%	NA	NA	NA	Development Testing Validation
Hossain et al.	NA	Before feature selection DT = 73.3%; RF = 94.4%; Adaboost = 94.4%; k‐NN = 68.7%; LR = 93.3%; NB = 93.1%; ANN = 74.4% After feature selection DT = 88.9%; RF = 95.6%; Adaboost = 95.6%; k‐NN = 90.1%; LR = 94.4%; NB = 84.7%; ANN = 92.2%	NA	Before feature selection DT = 97.4%; RF = 95.4%; Adaboost = 94.6%; k‐NN = 68.7%; LR = 91.6%; NB = 93.1%; ANN = 75.0% After feature selection DT = 89.3%; RF = 95.4% Adaboost = 93.6% k‐NN = 91.6%; LR = 91%; NB = 84.7%; ANN = 91.6%	Before feature selection DT = 100%; RF = 98.8%; Adaboost = 98.8%; k‐NN = 47.2%; LR = 94.4%; NB = 93.4%; ANN = 75.6% After feature selection DT = 95.2%; RF = 97.7%; Adaboost = 97.7%; k‐NN = 92.5%; LR = 92.4%; NB = 89.74%; ANN = 95.40%	NA	Before feature selection DT = 78%; RF = 99%; Adaboost = 99%; k‐NN = 52%; LR = 98%; NB = 98%; ANN = 83% After feature selection DT = 95%; RF = 99%; Adaboost = 99%; k‐NN = 97%; LR = 98%; NB = 98%; ANN = 98%	NA	NA	NA	Development Testing Internal validation—Cross‐validation folds
Hu et al.	NA	NA	GA + SSAE = 100%; RF + BP = 89.1%	GA + SSAE = 100%; GA + BP = 98%; GA + ELM = 98%; ReliefF + SSAE = 98%	NA	GA + BP = 97.1% GA + ELM = 97.1% GA + SSAE = 100%	GA + SSAE = 100%; ReliefF + SSAE = 100%; GA + ELM = 97.8%; GA + BP = 97.8%	NA	NA	NA	Development
Kinar et al.	NA	25%	NA	NA	NA	NA	NA	NA	NA	NA	Testing
Lindvall et al.	NA	Token‐level BERT‐base = 86% ClinicalBERT = 86% XLNet‐base = 88% ClinicalXLNet = 84% DistilBERT‐base = 86% RoBERTa‐base = 86% XLMRobERTa‐base = 88% ELECTRA‐small = 87% ClinicalELECTRA‐small = 80% Longformer‐base = 77%	NA	NA	Token‐level BERT‐base = 89% ClinicalBERT = 89% XLNet‐base = 84% ClinicalXLNet = 87% DistilBERT‐base = 87% RoBERTa‐base = 88% XLMRobERTa‐base = 88% ELECTRA‐small = 86% ClinicalELECTRA‐small = 77% Longtormer‐base = 89%	Token‐level BERT‐base = 87% ClinicalBERT = 88% XLNet‐base = 86% ClinicalXLNet = 85% DistilBERT‐base = 87% RoBERTa‐base = 87% XLMRobERTa‐base = 88% ELECTRA‐small = 87% ClinicalELECTRA‐small = 78% Longformer‐base = 82%	NA	NA	NA	NA	Testing
Lo et al.	NA	RF = 87.5%	RF = 87.2%	RF = 87.9%	NA	NA	RF = 94%	NA	NA	NA	Training
Malhotra et al.	NA	LR (age 15–60) = 72.5% (age 61–99) = 65.1%	LR (age 15–60) = 58.7% (age 61–99) = 56.8%	NA	NA	NA	LR (age 15–60) 65.6% (age 61–99) 60.9%	LR (age 15–60) 40% (age 61–99) 32.5%	LR (age 15–60) 72.2% (age 61–99) 83.6%	NA	Testing
Masadah et al.	Model 1 = 41% Model 2 = 41%	Model 1 = 70%; Model 2 = 60%	Model 1 = 88%; Model 2 = 94%	Model 1 = 82%; model 2 = 82%	NA	NA	Model 1 = 84%; Model 2 = 84%	NA	NA	NA	Testing Internal validation—Cross‐validation
Mezher et al.	NA	GFS = 96.2%	NA	GFS = 96.2%; RF = 95.8%; SVM (linear) = 93.6%; SVM (RBF) = 91%; SVM (polynomial); = 89.7%	NA	NA	GSF = 97%	NA	NA	NA	Development Internal validation—Cross‐validation folds
Mishra et al.	NA	GBFS‐Random forest model Lung cancer = 97.8% Skin cancer = 93.8% Cervical cancer = 97.4% Breast cancer = 96.8% Kidney cancer = 95.9% The mean sensitivity over cancer datasets = 96.3%	GBFS‐Random forest model Lung cancer = 97.5% Skin cancer = 92.8% Cervical cancer = 98.2% Breast cancer = 96.6% Kidney cancer = 96.2% The mean sensitivity over cancer datasets = 96.3%	GBFS‐Random forest model Lung cancer = 98.8% Skin cancer = 94.4% Cervical cancer = 98.4% Breast cancer = 97.4% Kidney cancer = 96.4% The mean accuracy over cancer datasets = 97%	NA	GBFS‐Random forest model Lung Cancer = 97.6% Skin Cancer = 93.5% Cervical Cancer = 97.9% Breast Cancer = 96.7% Kidney Cancer = 96% The mean F1 over cancer datasets = 96.3%	NA	NA	NA	NA	Training NA
Muhammad et al.	NA	DS3 Training set 87.3% Testing set 80.7%	DS3 Training set 80.8% Testing set 80.7%	NA	NA	NA	DS1 = 0.75 ± 0.06 (training sets); 0.71 ± 0.11 (testing sets) DS2 = 0.64 ± 0.01 (training sets); 0.62 ± 0.04 (testing sets) DS3 = 0.86 ± 0.01 (training sets); 0.85 ± 0.02 (testing sets)	DS3 Training set 0.1% (95% CI 0.09–0.100) Testing set 0.089% (95% CI 0.084–0.095)	DS3 Training set 99.997% (95% CI 99.996–99.997) Testing set 99.995% (95% CI 99.993–99.996)	NA	Training and Testing Internal validation—Cross‐validation folds
Mukherjee et al.	NA	NA	NA	SVM = 99.9%; MLPE = 99.6%	NA	SVM = 100%; MLPE = 100%	SVM = 100%; MLPE = 100%	SVM = 99.8%; MLPE = 99.6%	SVM = 100%; MLPE = 98.5%	NA	Training Internal validation—Cross‐validation folds
Nemlander et al.	NA	Never smoker = 70% Former smoker = 82% Current smoker = 83%	Never smoker = 88% Former smoker = 33% Current smoker = 62%	Never smoker = 82% Former smoker = 63% Current smoke = 77%	NA	NA	Never smoker = 74% Former smoker = 60% Current smoker = 82%	Never smoker = 78% Former smoker 67% Current smoker 85%	Never smoker = 83% Former smoker = 53% Current smoker = 57%	NA	Training Internal validation—Cross‐validation folds
Nemlander et al.	NA	73%	84%	NA	NA	NA	83%	NA	NA	NA	Training Internal validation—Cross‐validation folds
Oliver et al.	NA	RF = 88%; RT = 77%	NA	94.3%	RF = 71%; RT = 77%	RF = 79%; RT = 74%	RF = 73%; RT = 67%	NA	NA	NA	Training Internal validation—Cross‐validation folds
Rajan et al.	NA	NA	NA	MNN = 100%	NA	NA	NA	NA	NA	NA	Development NA
Senturk et al.	NA	GBT = 63.2%; k‐NN = 50%; RF = 71.5%; SVM = 100%; ANN = 100%	NA	GBT = 80%; k‐NN = 81.8%; RF = 81.5%; SVM = 100%; ANN = 100%	NA	NA	NA	NA	NA	NA	Training and Testing NA
Wani et al.	NA	99%	NA	98%	NA	98%	NA	NA	NA	NA	Training Internal validation—Cross‐validation folds
Weegar et al.	NA	BNB = 45%–85%	RF, SVM > 97%; BNB (62%; −92%); CNB (89%; −91%)	NA	RF = 53%–92%	NA	RF = 70%	NA	NA	NA	Training and Testing Internal validation—Cross‐validation folds
Xie et al.	NA	NA	NA	NA	NA	NA	Full model = 84% (95% CI 81–87) Simple model = 82% (95% CI 78–85)	NA	NA	NA	Development Internal validation—Cross‐validation folds
Zadsafar et al.	NA	NA	NA	ANN = 100%; Diff+k‐NN = 99.7%; SGD = 100%; SVM = 98%; GA + SSAE = 100%; BHHO+DT = 100%	ANN = 100%; SGD = 100%; SVM = 94.50%; GA + SSAE = 100%; BHHO + DT = 100%	SGD = 100%; GA + SSAE = 100%; BHHO + DT = 100%	NA	NA	NA	NA	Training and Testing NA

Abbreviations: AUC, Area under curve; BP, Backpropagation Algorithm; BNB, Bernoulli Naive Bayes; BHHO, Binary Harris Hawk optimization; CC, Clojure Classifier; CNB, Complement Naive Bayes; DS, Dataset; DT, Decision Tree; ANN, Deep Learning Artificial Neural Network; XGBoost, Extreme Gradient Boosted Decision Tree; ELM, Extreme Learning Machine; GA, Genetic algorithm; GFS, Genetic Folding Strategy; GBFS, Gradient Boosted Feature Selection‐Random forest model; GBT, Gradient booster trees; VFDT, Hoeffding Tree; KLR, Kernel Logistic Regression; k‐NN, k‐Nearest Neighbor; LDA, Linear Discriminant Analysis; LR, Logistic Regression; MLP, Multilayer Perceptron; MLPE, Multilayer Perceptron Ensemble; NB, Naive Bayes; PBNN, Perceptron‐based neural network; s‐Pegasos, Primal Estimated Sub‐gradient Solver for Support Vector Machine; PNN, Probabilistic neural network; RF, Random Forest; SSAE, Stacked Sparse Autoencoder; SGD, Stochastic gradient descent; SVM, Support Vector Machine; VP, Voted perceptron.

In terms of prediction horizon, the only studies that reported this information (*n* = 6) [42, 52, 61, 64, 66, 69] focused on predictions of cancer within a 5‐year timeframe.

#### 3.4.1 Performance Comparison Between Traditional and ML‐Based Predictive Models

Some studies [40, 42, 43, 45, 48, 49, 51, 52, 64] compared the predictive capabilities of ML with conventional methods. Specifically, in one study [40] the comparison was made between several ML models and the current UK esophagogastric cancer risk‐assessment tool (ogRAT). Importantly, both the ML models and the ogRAT were developed and validated using the same dataset, derived from the UK General Practice Research Database (GPRD). This ensures a fair comparison, as the models were evaluated on identical datasets. The study used symptoms encoded as binary variables (presence/absence) and included recurrence for specific symptoms (e.g., dyspepsia, dysphagia). Symptoms were parameterized individually, with no explicit pairwise or higher‐order combinations analyzed in the models. The ML models achieved similar performance, with an accuracy of 0.89 (95% CI: 0.86–0.92) and an AUROC of 0.87 (95% CI: 0.84–0.90), compared to the ogRAT's AUROC of 0.81 (95% CI: 0.79–0.83). The ML models identified 11% more cancer patients than the ogRAT, with minimal impact on false positives, or up to 25% more patients with a slight increase in false positives depending on the decision threshold. Symptoms were parameterized using binary encoding to represent the presence or absence of each symptom, with repeated occurrences recorded for certain symptoms. No further parameterization was applied to analyze combinations of symptoms, either pairwise or higher order.

In another study [43], an ML‐based symptomatic assessment chatbot was evaluated for its ability to identify women with breast cancer. The chatbot utilized an NLP algorithm combined with decision trees to analyze patient‐reported symptoms and risk factors. The model was compared against benchmark breast cancer assessment scores obtained from specialist doctors. The study reported that the chatbot achieved very high accuracy, with a sensitivity of 0.98 and specificity of 0.90, showing comparable performance to the specialists' scores. However, the specific dataset used for training and testing the chatbot was not identical to the data used by the specialists, as the chatbot relied on standardized inputs, such as symptom descriptions and risk factor data, while doctors had access to broader clinical information. This discrepancy made the comparison between the chatbot and human specialists less direct, and no 95% confidence intervals or false positive rates were reported for the chatbot's performance. Symptoms were parameterized using a weighted scoring system (1–10), reflecting the seriousness of responses, as defined by breast cancer specialists. While individual symptoms were explicitly scored, the study did not address pairwise or higher‐order symptom combinations. The chatbot's rule‐based design focused on single symptom assessment per query, with the knowledge base structured to guide sequential interactions rather than explore symptom interactions.

Dirik [45] compared ML models, including LR, for lung cancer diagnosis based on 15 binary symptoms (e.g., persistent cough, chest pain, weight loss). LR achieved 86% accuracy, while NB and SVM outperformed it with 91%. Performance metrics such as sensitivity, specificity, and precision confirmed the higher reliability of ML models. Symptoms were parameterized as binary variables (presence/absence), with no analysis of pairwise or higher‐order symptom combinations. While the paper demonstrated that ML models performed better than LR, it does not provide detailed reasons for this or explore differences in feature handling between methods.

Another study [42] compared models for predicting nasopharyngeal carcinoma, with LR as the baseline achieving an AUROC of 0.80. ML models, particularly LGB, outperformed LR, reaching an AUROC of 0.83 with higher sensitivity and specificity. The study incorporated 14 features, including demographics, 28 pre‐NPC symptoms, and combined diagnostic and treatment features, all parameterized as binary variables. Although combined features were used to improve accuracy, the study did not investigate pairwise or higher‐order symptom combinations, limiting the analysis of symptom interactions. Similarly, another study [48] compared ML models, including LR, for predicting endometrial intraepithelial neoplasia and endometrial cancer risks. LR served as the baseline but was outperformed by the MLP model, which achieved the highest AUC of 0.938 for precancer prediction. The study utilized 9 features, including age, BMI, and endometrial thickness, parameterized as continuous variables, with the Boruta algorithm selecting the most important features. Pairwise or higher‐order symptom combinations were not analyzed explicitly, as ML models focused on individual predictors.

Two further studies reported the superiority of ML models over conventional diagnostic techniques, evaluating their performance based on economic viability, practical implementation, and non‐invasiveness. Specifically, Muhammad et al. [66] developed a non‐invasive ML model for endometrial cancer risk stratification using demographic and clinical data, aiming to reduce patient burden and healthcare costs by avoiding invasive procedures like biopsies. However, no confidence intervals were reported, and the model was not directly compared with traditional methods. Gorynski et al. [63] developed an ML model for the early diagnosis of nasopharyngeal carcinoma, achieving an AUROC of 0.998% and 97.9% accuracy, though without confidence intervals or external validation. Both ML approaches showed advantages in terms of cost‐effectiveness and non‐invasiveness.

#### 3.4.2 Performance Comparison of Different ML‐Based Predictive Models

Most of the included studies (*n* = 22) [39, 40, 42, 45, 46, 47, 48, 49, 50, 51, 52, 53, 54, 59, 61, 63, 64, 65, 67, 68, 69, 70] compared various different ML‐based predictive models. In one study [62], which developed a model for mesothelioma diagnosis using the Harris Hawk Optimization (HHO) algorithm for feature selection, SVM and ANN both achieved 100% accuracy when trained on a set of 17 selected variables. This was compared to other methods such as DE with KNN, which achieved 99.7% accuracy (95% CI not provided) in the development, and a standard SVM model, which achieved 98% accuracy using 33 features. The models utilizing the HHO‐selected features demonstrated superior performance by optimizing the feature set, reducing redundancy, and enhancing diagnostic accuracy. However, no validations of these models were carried out.

GBFS and GFS demonstrated excellent performance in validation, with accuracy rates of 97.8% (95% CI: 96.4%–98.7%) and 96.2% (95% CI: 94.8%–97.6%), respectively, using fewer variables (16 and 15). SAC reached an AUC of 95% (95% CI: 93.0%–96.5%) with 16 variables. Several other models, including RF, Adaboost, LR, and NB, displayed accuracies between 93.1% and 94.4%, all using 16 variables. However, confidence intervals for these models were not consistently reported, limiting the interpretability of the results.

Another study that used the same dataset and variables to compare the performance measures of various ML models was conducted by Hossain et al. [49]. In this study, all models were trained and tested on the same dataset, consisting of 16 symptom‐based variables collected from 840 patients, including both leukemia and non‐leukemia cases. The performance metrics reported were based on the validation set, following a train‐test split where data from one hospital was used for training and data from another hospital was used for validation. The DT model achieved the highest performance, with an accuracy of 97.45%, an AUC of 0.783, and an MCC of 0.63. The RF model followed closely, with an accuracy of 95.41% and an AUC of 0.782. AB showed strong results, with an accuracy of 94.66%, while NB reached an accuracy of 93.13%, and LR achieved 91.60%. The k‐NN model exhibited the lowest performance, with an accuracy of 68.70%. Confidence intervals were not reported for these models.

A study [68] reported relevant differences in performance across various ML models, using a dataset of 324 patient records and clinical variables. All models were trained and tested on the same dataset, with performance metrics reflecting the results from the test sets, following an 80/20 random split for training and testing. RF achieved the highest performance, with a Matthews Correlation Coefficient (MCC) of +0.37, a specificity of 0.97, and a sensitivity of 0.28, using all 33 variables. MLP obtained an MCC of +0.11, with a sensitivity of 0.66 and a specificity of 0.42, also utilizing the full dataset. DT, applied only to the selected features of lung side and platelet count (2 variables), resulted in an MCC of +0.28, a specificity of 0.95, and a sensitivity of 0.28. The One Rule model demonstrated an MCC of +0.27, with a specificity of 0.97 and a low sensitivity of 0.17, using all 33 variables. No confidence intervals were reported for these performance metrics. Specific performance data are reported in Table 4.

Another study [39] demonstrated that a classifier model based on super symptom analysis performed better than classical methods such as ANN, LDA, and RF in terms of accuracy for breast cancer diagnosis. In this study, the models were trained and tested using the same dataset of 65 breast cancer patients, with a 70/30 split for training and validation. The super symptom analysis model achieved the highest accuracy at 72.7%. In comparison, ANN achieved an accuracy of 45.4%, LDA reached 57.1%, and RF achieved 63.1%. The study did not provide confidence intervals for these performance metrics. All models used the same variables and were evaluated on the validation set, allowing for a consistent comparison of their effectiveness in diagnosing breast cancer.

### 3.5 Quality Assessment of Included Studies: QUADAS‐AI

In the included studies, adherence to established reporting guidelines was not frequently mentioned. Out of the 34 studies, none explicitly acknowledged their adherence to the Transparent Reporting of a multivariable prediction model for Individual Prognosis or Diagnosis (TRIPOD) reporting guidelines [37]. Additionally, while making implementation codes publicly accessible is important for transparency and reproducing methods, only 5 [40, 49, 50, 60, 68] studies made their analysis code publicly available.

**FIGURE 4:**
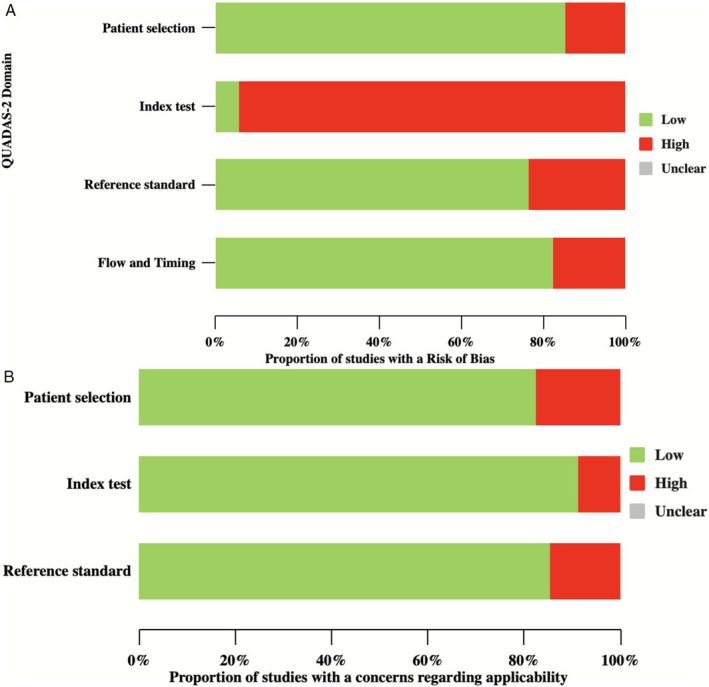
Proportion of studies with (A) risk of bias and (B) concerns regarding applicability.

A detailed analysis of the risk of bias assessment and concerns regarding applicability was performed for each study and summarized in Figure 4A,B. *Risk of bias*: 5 studies (14.7%) [50, 55, 60, 70, 71] had a high risk of bias in patient selection, mostly due to the lack of a clear rationale for their sample size and an unspecified data source. Most of the studies had a high risk of bias for the index test (94.1%), typically due to the lack of validation or testing on external data. In the reference standard domain, which assesses whether the method used as the gold standard for diagnosis or outcome measurement is reliable and applied appropriately, 76.5% of articles were at a low risk of bias. Finally, the risk of bias in the flow and timing domain was low in 82.4% of studies. *Applicability concerns*: In the patient selection domain, concerns about applicability were high in 17.6% [45, 50, 55, 60, 70, 71] of the included studies. In the index test domain, concerns about applicability were low in 91.2% of the studies due to the lack of detail on the construct or architecture of the algorithm. Finally, in the reference standard domain, concerns about applicability were low in 85.3% of studies. The main reasons for lower applicability included the use of surrogate or proxy measures instead of gold‐standard diagnostic tests, reliance on internal validation only (with only two studies [42, 50] explicitly performing external validation), and patient populations that may not be representative of the broader clinical setting. Additionally, issues like improper patient flow, where inclusion or exclusion criteria are unclear, or where the patient population does not mirror real‐world clinical practice, could introduce bias and further reduce applicability.

## 4 Discussion

This systematic review summarizes the reported potential of different types of ML models for predicting cancer risk based on clinical signs, symptoms, and other patient characteristics. The reviewed evidence indicates that ML models demonstrated variable performance, with AUC values ranging from 0.60 to 1 during validation. This variability reflects differences in dataset quality, feature selection methods, and model optimization techniques across studies. Models such as RF, SVM, and DT were often reported to achieve high accuracy, particularly in lung cancer and mesothelioma prediction. However, variability in performance highlights the need for further evaluation to determine their robustness and suitability for clinical use.

A growing interest in using ML models for cancer risk prediction is apparent, fueled both by recent advancements in ML technology and the increasing focus on precision medicine in supporting clinical decision‐making [72, 73, 74].

The reviewed studies used a broad range of data types, including symptoms, sociodemographic characteristics, lifestyle factors, comorbidities, genetic information, laboratory, and diagnostic test results. This diversity of input data emphasized the flexibility of ML models to integrate multiple sources of information, potentially improving the accuracy of cancer risk predictions by creating a more comprehensive risk profile for each patient. On the other hand, the variability in the quality of data across studies may also explain the wide range of model performance. Studies incorporating genetic data (although used in only 8 studies [43, 47, 48, 49, 52, 55, 63, 66]) or laboratory results (*n* = 9) [38, 40, 46, 59, 62, 63, 65, 68, 70], in addition to symptoms, could provide more nuanced risk assessments [43] underscoring the importance of multimodal data integration in enhancing predictive capabilities. However, models with too many variables risk overfitting, particularly when the data is limited or highly specific to the training population. This highlights the need for careful selection and optimization of variables to balance model complexity with robustness [75]. Moreover, for ML algorithms to be effectively deployed in clinical practice, they need to be trained and tested on datasets that adequately represent the clinical scenarios likely to be encountered in real‐world settings [76, 77, 78, 79]. This ensures that the range and depth of variables used to develop the model—such as symptoms, diagnostic tests, and comorbidities—are also accessible in the clinical environment where the model will be implemented. The clinical relevance and applicability of AI models depend not only on the representativeness of the training data but also on the alignment between the data infrastructure available during model deployment and the wealth of variables utilized during model development [80, 81]. This highlights the importance of a robust IT support system in hospitals or clinics to enable the integration of these models into a “learning health system” framework. Furthermore, a clear and transparent description of the training data characteristics—such as how data were collected, labeled, and processed—is critical to identifying potential sources of bias and ensuring clinical applicability [82]. Despite this, there is currently no standardized approach within the medical community for documenting datasets used in AI model development, leading to calls for greater transparency in the field [83].

From a clinical perspective, ML‐based cancer risk prediction models have the potential to support earlier diagnosis, guide surveillance strategies, and enable more personalized patient management. For clinicians, these tools may assist in identifying high‐risk individuals who could benefit from intensified monitoring or preventive interventions, even when conventional risk factors [84] are absent or unclear. For patients, particularly those in underserved or high‐risk populations, such models may facilitate more equitable access to timely risk assessment and targeted care pathways. By focusing exclusively on models that included at least one symptom or sign as a predictor, this review encompasses the subset of ML tools most relevant as a clinical decision support for symptomatic patients. Consequently, our findings mainly reflect the potential of ML in supporting clinical diagnostic decision‐making in symptomatic presentation settings, rather than across the full cancer risk continuum.

A limitation identified in this review is the lack of transparency regarding the interaction and contribution of these modalities in final predictions. While fully achieving interpretability in black‐box models remains challenging, the integration of post hoc explainability methods, such as SHapley Additive exPlanations (SHAP) or Local Interpretable Model‐agnostic Explanations (LIME), could provide valuable insights into the relative influence of genetic, clinical, and sociodemographic factors. This added transparency, even if partial, could improve the clinical relevance and trustworthiness of these predictive models. Traditional statistical methods, though limited in automatic variable selection, allow for explicit definitions of non‐linear associations and interactions, encouraging thoughtful model construction [85]. In contrast, ML models automatically capture complex relationships, which may simplify modeling but also risk bypassing important contextual insights. This distinction is critical, as prediction models capture correlations that do not imply causation [86]. Cautious interpretation is therefore essential; explainable models might reveal feature importance without clarifying causal links, underscoring the need for clinicians to interpret machine learning‐generated risk predictions carefully, ensuring clinical decisions are not misinformed by correlations alone.

Most studies using restricted access data did not describe important demographic information, and most models were not available for additional evaluation to assess robustness. The lack of details and transparency about data and models limited our ability to systematically assess robustness and potential biases, which is an important direction for future work. Another key issue is algorithmic bias, which arises when models are trained on unrepresentative data, leading to inaccurate predictions for certain groups. This can worsen healthcare disparities. Developers must address this by using diverse datasets, evaluating model performance across demographics, and correcting biases to ensure fair and equitable healthcare outcomes [87, 88, 89].

External validation of a risk model is critical for ensuring trustworthiness before clinical deployment [90, 91, 92]. Unfortunately, in this review, only two of the 35 included studies conducted an external validation. This is an important limitation, as without proper external validation, it is difficult to determine whether the predictive performance of the models would hold up when applied to diverse patient populations or different healthcare environments. One [50] of the two studies that conducted external validation focused on using unstructured clinical notes from electronic health records to extract cancer‐related symptoms. This study developed a DL model to automatically identify symptoms documented in free‐text format, which is challenging to process at scale. The authors first trained the model using outpatient notes and then performed external validation using the MIMIC‐III dataset, which consists of physician notes from intensive care units. In this external validation, the model continued to perform well, with F1 scores ranging from 0.97 for symptoms like diarrhea and dizziness to 0.73 for swelling. These results indicate that the model was successful in capturing a wide range of clinically significant cancer‐related symptoms both in the original training setting and in a different clinical context, making it a valuable tool for scalable symptom monitoring in oncology.

The utilization of unstructured data is an emerging trend in the field of ML for healthcare. Castro et al. [93] explored the potential of ML in predicting breast cancer recurrence by combining structured and unstructured healthcare data. Tayefi et al. [94] explored the use of electronic health records for healthcare, addressing the challenges and potential of integrating structured and unstructured data. Deshmukh et al. [95] developed a clinical decision‐unifying staging method to accurately extract and predict the prognostic stage of breast cancer from unstructured medical records across various health institutions.

The comparison between ML models and traditional statistical models for cancer risk prediction suggests that ML may offer advantages in handling complex, nonlinear relationships within high‐dimensional datasets, as it does not require manually specifying the functional form of these relationships in the model. Despite these advantages, traditional models like ogRAT benefit from extensive external validation, which has proven their reliability in real‐world clinical settings and simplicity. In contrast, a relevant number of ML models, including those previously mentioned, still require robust external validation to guarantee their generalizability and consistency across diverse patient populations and healthcare systems. Further efforts in external validation are crucial for their broader adoption in clinical practice.

A notable finding of this review is that none of the included studies explicitly adhered to the TRIPOD reporting guideline. This represents a major limitation in the methodological transparency of the current literature. Without adherence to standardized reporting frameworks such as TRIPOD or the forthcoming TRIPOD‐AI, key details regarding model development, variable selection, validation strategy, and calibration are often omitted, hindering reproducibility and independent assessment of model quality. The absence of structured reporting not only limits interpretability and external validation but also impedes the integration of these models into clinical practice. Future research should prioritize compliance with TRIPOD‐AI to ensure clarity, transparency, and comparability across ML‐based cancer risk prediction studies.

### 4.1 Limitations

This review has some limitations. First, only studies published in English were included, as multilingual screening, study assessment, and data extraction were not feasible within the research team. This may have led to the exclusion of relevant non‐English studies. Second, we focused on peer‐reviewed literature and did not include gray literature, preprints, theses, or conference proceedings. This approach ensured methodological rigor and reproducibility, but it might have limited the capture of emerging AI‐based research given the fast pace of developments in this field.

A major limitation of this review, and of the literature it synthesizes, is the sparsity of detailed reporting in many of the included studies, which restricted our ability to fully evaluate essential aspects such as model calibration and reproducibility. In addition, an important limitation concerns the high risk of bias observed in the index test domain, primarily due to the lack of external validation. Only 2 out of 35 studies conducted external validation, and even these were often limited to retrospective datasets from similar institutional settings. For example, while Lindvall et al. [50] validated a deep learning model on ICU notes, it was not tested in a primary care setting where the majority of symptomatic patients initially present. This severely limits the generalizability and clinical applicability of the reviewed models. Therefore, the conclusions should be interpreted with caution.

The included studies were highly heterogeneous in terms of data sources, cancer types, and predictive model architectures, which limited the possibility of direct comparison and meta‐analysis. Although all studies incorporated symptoms, the type and combination of additional predictors, such as sociodemographic characteristics, comorbidities, and laboratory or genetic data, varied substantially, contributing to the variability in model performance. This heterogeneity also influenced the assessment of methodological quality. The high proportion of studies rated as having a high risk of bias within the index test domain mainly reflects incomplete reporting, lack of external validation, and limited methodological transparency, rather than uniform methodological flaws. While alternative thresholds or subgroup analyses (for instance, stratified by study design, publication year, or validation method) might slightly modify the distribution of bias ratings, the overall conclusion remains consistent: current ML‐based cancer risk prediction studies display considerable methodological diversity and suboptimal reporting standards, highlighting the need for more rigorous and standardized research in this field.

Furthermore, our review highlights a recurring limitation in reporting performance metrics across primary studies. While AUC was the most frequently reported measure, it does not fully capture clinical utility. Only a minority of studies provided additional metrics such as calibration, positive predictive value, or net benefit, key elements for assessing real‐world applicability. Future research should prioritize comprehensive and standardized reporting of clinically relevant metrics to support the safe and effective translation of ML models into practice.

Another factor that may limit generalizability is the geographic distribution of the included studies, which was heavily skewed toward Asian countries. While this reflects the rapid integration of AI technologies and availability of large‐scale clinical datasets in these settings, differences in healthcare infrastructure, diagnostic practices, and population characteristics may restrict the applicability of these models to other regions. Future research should aim to validate and adapt ML‐based cancer risk prediction models across diverse healthcare systems to enhance external validity and global relevance.

Limited information on variable selection and data sources further restricted our assessment of model robustness. Moreover, the review focused only on indexed articles, potentially overlooking valuable insights from preprints and conference proceedings that are prominent in AI research but not yet peer‐reviewed or included in databases like PubMed, which may have narrowed our findings. Inconsistent approaches to handling missing data, validation, and variability in reporting standards across studies made it difficult to compare models and assess their practical applicability.

Another limitation is the focus on longer prediction horizons, which restricts the evaluation of model performance in shorter‐term contexts, such as 6‐month predictions. Longer follow‐up periods allow for higher predictive accuracy, as events such as cancer development are more likely to occur over longer periods. Of the included studies, only six [40, 50, 59, 62, 64, 67] reported prediction horizons, all within a 5‐year window. Short‐term predictions are more challenging but are critical for timely clinical decision‐making and early intervention. Future research should explore the performance of ML models over shorter prediction intervals to address this gap and enhance their practical utility in urgent clinical scenarios.

Additionally, this study could not determine whether the comparisons between ML models and conventional statistical techniques were entirely appropriate. In many instances, the statistical comparator used may not reflect the optimal implementation of traditional methods, potentially biasing results in favor of ML models. This raises concerns about the rigor and validity of such comparisons, as suboptimal statistical models may not provide a robust benchmark for evaluating ML performance. Greater transparency and standardization in the design and reporting of both ML and conventional models are essential to ensure fair and meaningful head‐to‐head evaluations.

Also, this review may be subject to selection bias. We excluded non‐English language studies and restricted our search to indexed literature, thereby potentially overlooking relevant gray literature and studies published in other languages. While these criteria ensured the inclusion of peer‐reviewed and methodologically sound sources, they may have narrowed the scope of the findings, especially in the context of a rapidly evolving research area such as machine learning in cancer prediction.

Finally, a key limitation of the reviewed studies is the lack of implementation of models within a learning health system framework. Such systems are critical for evaluating the real‐world performance and impact of predictive models, as they enable continuous feedback, adaptation, and integration into clinical workflows. Without this framework, it remains challenging to assess how these models perform in dynamic healthcare settings, further emphasizing the need for future research to embed predictive models into learning health systems to ensure their practical utility and scalability.

## 5 Conclusions

ML models show promise in managing high‐dimensional data and capturing complex, non‐linear relationships, but their practical applicability in clinical settings remains to be fully established. Techniques such as LR with carefully selected interaction terms continue to demonstrate reliability and utility. Achieving a balance between model complexity, interpretability, and clinical relevance is crucial for improving practical applicability in healthcare. Key methodological challenges, including limited external validation and calibration issues of ML models, must be addressed to enable their clinical adoption. Enhancing reporting practices—through thorough documentation of data preprocessing, model training, and validation—will be essential for developing robust and clinically useful risk prediction tools. Further research is needed to robustly compare the diagnostic accuracy of ML models and traditional statistical methods, with a focus on explaining performance differences and considering the influence of prediction horizons. Additionally, implementation studies evaluating these algorithms within learning health systems are critical to determine their real‐world applicability and impact. Future research should prioritize adherence to TRIPOD‐AI guidelines, transparent sharing of code and data, and standardized reporting of prediction horizons, missing data handling, and calibration metrics. Embedding ML models within learning health systems and ensuring fairness across diverse populations will be essential steps to enhance their real‐world utility, reliability, and ethical deployment in oncology.

## Author Contributions

**Flavia Pennisi:** conceptualization, investigation, writing – original draft, methodology, validation, visualization, writing – review and editing, data curation, formal analysis, software. **Stefania Borlini:** investigation, writing – original draft, visualization, data curation, software. **Hannah Harrison:** writing – review and editing, methodology, supervision. **Rita Cuciniello:** investigation, visualization, formal analysis, writing – original draft, software. **Anna Carole D'Amelio:** writing – original draft, visualization, data curation, software. **Matthew Barclay:** writing – review and editing, methodology, formal analysis. **Giovanni Emanuele Ricciardi:** visualization, writing – review and editing. **Georgios Lyratzopoulos:** supervision, methodology, writing – review and editing. **Cristina Renzi:** conceptualization, writing – review and editing, writing – original draft, resources, supervision, funding acquisition, project administration.

## Funding

Prof Cristina Renzi and Prof Georgios Lyratzopoulos were funded by the early detection and diagnosis committee grant EDDCPJT\100018 from Cancer Research UK. Prof Cristina Renzi, Prof Georgios Lyratzopoulos, dr Flavia Pennisi and dr Giovanni Emanuele Ricciardi were founded by grant May24/100066 from Cancer Research UK.

## Disclosure

Role of the funder/sponsor: The funders had no role in the design and conduct of the study; collection, management, analysis, and interpretation of the data; preparation, review, or approval of the manuscript; and decision to submit the manuscript for publication.

## Consent

The authors have nothing to report.

## Conflicts of Interest

The authors declare no conflicts of interest.

## Supporting information


**Table S1:** Literature search strategy (Pubmed).
**Table S2:** Data information of included studies.
**Table S3:** Risk predictors.
**Table S4:** Risk predictors: specific variables included in “other”.
**Table S5:** Classification of included studies according to the types of data used in machine learning models for cancer risk prediction. A study was classified as “Included” for a given data type if that type was explicitly used as input in the final model. Studies integrating three or more data types (excluding symptoms, which were present in all studies) are defined as multimodal and are shown in bold.

## Data Availability

The data that support the findings of this study are available from the corresponding author upon reasonable request.
