# Supplementary material for: Cancer Risk Prediction Using Machine Learning for Supporting Early Cancer Diagnosis in Symptomatic Patients: A Systematic Review of Model Types
Source: Cancer Med. 2025 Dec 13;14(24):e71463. doi: 10.1002/cam4.71463 (PMC12701559; doi:10.1002/cam4.71463)
Supplement: Supplementary file 1 — Table S1: Literature search strategy (Pubmed). Table S2: Data information of included studies. Table S3: Risk predictors. Table S4: Risk predictors: specific variables included in “other”. Table S5: Classification of included studies according to the types of data used in machine learning models for cancer risk prediction. A study was classified as “Included” for a given data type if that type was explicitly used as input in the final model. Studies integrating three or more data types (excluding symptoms, which were present in all studies) are defined as multimodal and are shown in bold. [file CAM4-14-e71463-s001.docx]

**Supplementary Table 1 -**  *Literature search strategy (Pubmed).*

| **Database** | **Search Date** | **Search String** | **Notes** |
| --- | --- | --- | --- |
| PubMed | from 1 January 2014 to 17 May 2024 | ("artificial intelligence"[All Fields] OR "AI"[All Fields] OR "machine learning"[All Fields] OR "deep learning"[All Fields] OR "neural network*"[All Fields] OR "support vector machine*"[All Fields] OR "Bayesian network"[All Fields] OR "nearest neighbour"[All Fields] OR "decision tree"[All Fields] OR "random forest"[All Fields] OR "binomial model"[All Fields] OR "probabilistic model"[All Fields] OR "conventional neural network"[All Fields] OR "Transfer Learning"[All Fields] OR "statistical model"[All Fields] OR "two-parameter model"[All Fields] OR "polynomial model"[All Fields] OR "Ensemble Learning"[All Fields] OR "risk prediction model*"[All Fields] OR algorithm*[All Fields])  AND  (cancer*[All Fields] OR tumour*[All Fields] OR tumor*[All Fields] OR neoplasm*[All Fields] OR malignan*[All Fields] OR oncology*[All Fields] OR carcinoma*[All Fields] OR sarcoma*[All Fields] OR melanoma*[All Fields] OR leukaemia[All Fields] OR leukemia[All Fields] OR lymphoma[All Fields] OR myeloma[All Fields])  AND  ("predict*"[All Fields] OR "assessment"[All Fields] OR "risk"[All Fields] OR "risk stratification"[All Fields] OR "clinical risk assessment"[All Fields] OR "risk prediction"[All Fields] OR "risk assessment model"[All Fields] OR "risk score"[All Fields] OR diagnos*[All Fields] OR recogni*[All Fields] OR identif*[All Fields] OR decision[All Fields] OR "predictive value*"[All Fields] OR "receiver operating characteristic curve*"[All Fields] OR "ROC curve*"[All Fields] OR "area under curve*"[All Fields] OR "area under the curve*"[All Fields] OR "AUC"[All Fields] OR "C statistic*"[All Fields] OR "discriminant*"[All Fields] OR "absolute risk*"[All Fields] OR "brier*"[All Fields] OR "lorenz curves"[All Fields])  AND  (symptom*[All Fields] OR "clinical risk factor*"[All Fields] OR "laboratory test*"[All Fields]) | English language articles |

**Supplementary Table 2** *- Data information of included studies*

| **Authors** | **Data source** | **Cancer sample size** | **Sample comparison or control** |
| --- | --- | --- | --- |
| Alam T.M. et al | Dataset of medical histories | NA | 228 |
| Al-Juboori F.S. et al | Cancer Oncology Hospital in Baghdad | 65 | NA |
| Bhuta P. et al | Sreenidhi Institute of Science and Technology, Hyderabad, Telangana, India dataset | 270 | 8 |
| Briggs E. et al | Electronic health record dataset derived from the UK General Practice Research Database | 7,471  Training set 75%  Testing set 25% | 32,877  Training set 75%  Testing set 25% |
| Chen A. et al | Hospital electronic medical records | 1,357  Training set 60%  Validation set 20%  Testing set 20% | 1,448  Training set 60%  Validation set 20%  Testing set 20% |
| Chen J.H. et al | Real-world FBC Q&A data obtained from a major hospital in Taiwan (Chang Gung Memorial Hospital) | 135 | NA |
| Chen J.W. et al | National Health Insurance Research Databases | Training set = 8,874  External validation =2,219  Testing set = 1 million | Training set = 8,874  External validation = 2,219 |
| Chen Q. et al | Optum electronic medical records data set | 3,322  Training set 70%  Testing set 30% | 53.15  Training set 70%  Testing set 30% |
| Chicco D. et al | Hospital electronic medical records | PNN= 96 PBNN =  Training set 60%  Validation set 20%  Testing set 20%  RF,DT =  Training set 80%  Testing set 20%  One rule =  Training set 80%  Testing set 20% | PNN= 228 PBNN =  Training set 60%  Validation set 20%  Testing set 20% RF, DT=  Training set 80%  Testing set 20% One rule=  Training set 80%  Testing set 20% |
| Choudhury A. et al | Dicle University, Turkey | 96 | 228 |
| Dirik M. et al | Survey | NA | 270 |
| Duan S. et al | First Affiliated Hospital of Zhengzhou University | 342 Training set  (280 bening and 62 lung cancer);  296 Testing set  (128 bening and 168 lung cancer) | NA |
| Erdemoglu E. et al | Department of Obstetrics and Gynecology, Suleyman Demirel University, Isparta, Turkey | 564 patients  Training set = 451  Validation set = 113  45 cases of precancer or endometrial cancer (7.9%) | NA |
| Goryński K. et al | Diagnostic and Monitoring of Tuberculosis and Illness of Lungs Ward in Kuyavia and Pomerania Centre of the Pulmonology (Bydgoszcz, Poland) | NA | 193  Training test = 97  Testing test = 48  Validating test = 48 |
| Hossain M.A. et al | National Institute of Cancer Research and Hospital (NICRH) as a testing set  Dhaka Shishu Hospital as a training set | Training set = 510  Testing set = 103 | Training set = 199  Testing set = 28 |
| Hu X. et al | University of California | 97  (Training set 70%  Testing set 30% | 227  Training set 70%  Testing set 30% |
| Kinar Y. et al | MHS electronic medical records from Israel Cancer Registry | NA | 3,337 |
| Lindvall C. et al | 1) the Dana-Farber Cancer Institute (**DFCI***) of the Epic Eletronic Health Reports (Verona, WI) 2) Medical Information Mart for Intensive Care III (**MIMIC-III****) data set of Beth Israel Deaconess Medical Center (Boston, MA) | Training set n° patients = 870 Testing set n° patients = 97  Training notes = 1,125  Test notes = 100 | NA |
| Lo C.L. et al | National Health Insurance Research Database (NHIRD) of Taiwan | 535 patients  (210 with delayed diagnosis,  325 with non-delayed diagnosis) | 210/535 |
| Malhotra A. et al | The English National Cancer Registry (CR) and Clinical Practice Research Datalink (CPRD) | NA | 4,556 |
| Masadah R. et al | medical records | Training set = 10  Testing set = 7 | Training set = 38  Testing set = 15 |
| Mezher M.A et al | Lung cancer dataset from Kaggle | 309  (39 benign and 270 malignant) | 39/309 |
| Mishra S. et al | UCI machine learning repository by the University of California | 488 | NA |
| Muhammad W. et al | National Health Interview Survey (NHIS) dataset and The Prostate, Lung, Colorectal, and Ovarian (PLCO) dataset  1. DS1=NHIS dataset (645,217 participants, with 131 PC cases)  2. DS2=PLCO dataset (154,897 participants with 767 PC cases)  3. DS3 = NHIS dataset + PLCO dataset (800,114 participants with 898 PC cases) | DS1= 131  DS2= 767  DS3= 898 Training set 70%  Testing set 30% | DS1= 645,217  DS2= 154,897  DS3= 800,114 Training set 70%  Testing set 30% |
| Mukherjee S. et al | hospital electronic medical records | NA | NA |
| Nemlander E. et al | Karolinska University Hospital | NA | 504 |
| Nemlander E. et al | Swedish Cancer Registry, VEGA regional administrative healthcare database (Primary health care data) | 542  Training set = 407  Testing set = 135 | 2,139  Training set = 1,606  Testing set =533 |
| Oliver A.S. et al | Data.world repository | Training set = 20 | Training set = 9 |
| Rajan et al | <https://data.world/cancerdatahp/lung-cancer-data> | NA | NA |
| Senturk Z.K. et al | Dicle University, Turkey | 96  Training set 80%  Testing set 20% | 228  Training set 80%  Testing set 20% |
| Wani N.A. et al | DataWorld, Survey lung cancer dataset | 309 | 309 |
| Weegar R. et al | Karolinska University Hospital | 1,321  Training set = 264  Testing set = 1,057 | 16,212  Training set = 3,242  Testing set = 12,970 |
| Xie S. et al | 195 hospital departments, six swedish oncological centers | 189 | 820 |
| Zadsafar F. et al | Dicle University | 96  Training set 80%  Testing set 20% | 228  Training set 80%  Testing set 20% |

**Supplementary Table 3** *- Risk predictors*

| **Authors** | **Demographics** | **Symptoms** | **Comorbidities** | **Genetic data** | **Lifestyle** | **Other*** | **Number of variables**  **included vs. considered** |
| --- | --- | --- | --- | --- | --- | --- | --- |
| Alam T.M. et al | Age  Gender  City | Dyspnea  Chest pain  Weakness | NA | NA | Smoking status | Asbestos exposure Clinical data  Blood count cell  Clinical biochemistry test  Pleural data | 26/35 |
| Al-Juboori F.S. et al | Age | Aspecific symptoms | NA | NA | NA | Cancer test  Cancer stage and type  Receptor status  Cell activity  Treatment and Condition | 8 |
| Bhuta P. et al | NA | Seizures  Headache  Nausea  Memory loss | NA | NA | NA | Type of treatment  Origin of tumor | 12 |
| Briggs E. et al | Age | Dysphagia  Epigastric pain  Weight loss  Dyspepsia  Reflux  Nausea and vomiting  Abdominal pain  Chest pain  Constipation | NA | NA | NA | Blood count cell  Clinical biochemistry test | 18/24 |
| Chen A. et al | NA | Nose, ear, eye,face,voice,throat symptoms  Bleeding (hemoptysis,epistaxis), Neck mass  Neck pain  Headache | Nasal and sinus conditions  Nasal sinus cyst  Sinusitis  Rhinitis  Nasopharyngitis  Pharyngitis  History of middle ear repairing or diseases  Lung/nose mass | NA | NA | NA | 20/163 |
| Chen J.H. et al | NA | Breast hurt  Breast lumps  Breast sunken  Bulged, or wounded  Breast skin itchy  Discharge from the nipple | Medical history of certain cancers:  Ovarian cancer  Breast cancer  Prostate cancer Pancreatic cancer | Family and genetic history  Family history of breast or ovarian cancer  Family history of metastatic prostate or pancreatic cancer  Genetic test positive for BRCA gene | NA | Imaging test  Breast health evaluations and procedures | 16/16 |
| Chen J.W. et al | Age  Gender | Nasal/aural symptoms  Headache  Bleeding ( hemoptysis, epistaxis)  Head and neck mass  Neurological sign | NA | NA | NA | Serum marker  Head and neck mass management | 14/64 |
| Chen Q. et al | Age  Gender  Education level, Income  Race | Jaundice  Abdominal pain  General pain  Anaemia | Biliary tract diseases Other liver diseases  Pancreatitis  Unspecified benign neoplasm  Disorders of lipid metabolism  Diabetes mellitus  Chronic kidney diseases  Hypertension  Other nervous system disorders | NA | NA | Pancreatic disorders (not diabetes)  Medication  Outpatient visits and diagnostic test | 582/18,220 |
| Chicco D. et al | Age  Gender  City | Duration of symptoms Dyspnea  Weakness  Ache on chest | NA | NA | Smoking status | Asbestos exposure  Blood test  Pleural data and fluid test  Imaging and functional test  Dead or not  Type of malignant Mesothelioma  Performance status | 33/35 |
| Choudhury A. et al | Age  Gender  City | Dyspnea  Chest pain  Weakness | NA | NA | Smoking status | Asbestos exposure  Blood count cell  Clinical biochemistry test | 35/NA |
| Dirik M. et al | Age  Gender | Yellow fingers  Fatigue  Wheezing  Cough  Dyspnea  Swallowing difficulties  Chest pain | Persistent lung infections  Anxiety  Allergy | NA | Smoking status  Drinking status | NA | 16/16 |
| Duan S. et al | Age  Gender | Chest tightness  Chest pain  Bloody sputum  Cough  Hemoptysis  Fever  Sweating | NA | Family history of lung cancer  History of lung infection  Family history of tumor | Smoking status  Drinking status | Biomarkers  Radiomic features | 14/41 |
| Erdemoglu E. et al | Age | Abnormal bleeding (Premenopausal/postmenopausal) | BMI  Hypertension  Diabetes mellitus | History of breast cancer | Smoking status | Diagnostic procedures  Surgical procedures | 3/9 |
| Goryński K. et al | Age  Gender  Education level  City | Weakness  Chest pain  Pneumonia  Weight loss  Cough  Lack of appetite  Fever  Bone pains  Dyspnea  Hemoptysis | BMI  Cardiovascular diseases  Other cancer  Chronic obstructive pulmonary diseases | Family history of lung cancer | Smoking status | Blood test  Pulmonary Function test  Imaging test | 47/48 |
| Hossain M.A. et al | NA | Dyspnea  Bone pain  Fever  Frequent infections Itchy skin  Rash  Loss of appetite  Nausea  Persistent weakness  Swollen painless lymph  Significant bruising Bleeding | NA | Family history of leucemia | Smoking status | NA | 16/16 |
| Hu X. et al | Age  Gender  City | Chest pain  Weakness | NA | NA | Smoking status | Asbestos exposure  Diagnostic information  Blood test  Pleural fluid test | 19/34 |
| Kinar Y. et al | Age  Gender | Gastrointestinal bleeding  Changes in bowel habits  Anaemia | Use of aspirin or other anticoagulants  Gastrointestinal illness Gastrointestinal cancer | NA | NA | Blood test | 9/NA |
| Lindvall C. et al | Age  Gender  Race | General pain  Anxiety  Fatigue  Sadness  Nausea  Neuropathy  Dyspnea  Insomnia  Constipation  Cough  Decreased appetite Diarrhea  Abdominal pain  Hoarseness  Headache  Swelling  Joint pain  Rash  Vomiting | NA | NA | NA | NA | 80/80 |
| Lo C.L. et al | Age  Gender  Hospital level  City | Hematuria | NA | NA | NA | Cystoscopy | NA/10 |
| Malhotra A. et al | NA | Abdominal pain  Constipation  Flatulence  Back pain  Pruritis  Insomnia  Weight loss  Fatigue  Anorexia  Anxiety  Weakness  Fever  Obesity  Disturbance in smelling/tasting  Odynophagia  Anaemia  Xerostomia  Stomatitis  Halitosis  Glossodynia  Polydipsia  Hyperlipidaemia  Dysmenorrhea | Cardiovascular diseases  Hypertension  Diabetes mellitus  Rheumatoid arthritis  Atopic diseases | Family history of breast cancer  Peutz-Jeghers syndrome  Family history of melanoma | Smoking status  Drinking status | Vascular conditions  Liver and gallbladder conditions  Gastrointestinal conditions  Drug-Related Issues  Kidney and Urinary Conditions  Gynecological conditions | 50/NA |
| Masadah R. et al | Age | Uterus bleeding | BMI | NA | NA | Abdominal enlargement  Biomarkers | 5/26 |
| Mezher M.A et al | Age  Gender | Yellow fingers  Anxiety  Peer pressure  Fatigue  Wheezing  Coughing  Dyspnea  Swallowing difficulty  Chest pain | Chronic diseases | NA | Smoking status  Drinking status | NA | NA/15 |
| Mishra S. et al | Age  Gender | Hemoptysis  Chest pain | Lung chronic risks  BMI | Gene disorders | Smoking status  Drinking status | NA | 16/16 |
| Muhammad W. et al | Age  Gender  Race | Asthma | Diabetes mellitus  Emphysema  Stroke  Cardiovascular diseases (coronary heart disease, angina pectoris, heart attack, other heart disease)  Hypertension  Ulcer  Other cancer | Family history of pancreatic cancer | Smoking status  Drinking status | NA | 18/18 |
| Mukherjee S. et al | Age  Gender  City | Duration of symptoms  Dyspnea  Chest pain  Weakness | NA | NA | Smoking status | Asbestos exposure  Diagnosis  Imaging and tests  Blood test  Pleural fluid test  Dead or not  Performance status | 34/34 |
| Nemlander E. et al | Age  Gender  Education level | Breathing worse upon exertion  Cough  Voice got hoarser  Felt cold  Wheezing  Feeling unwell  Appetite loss | NA | NA | Smoking status | NA | 10/73 |
| Nemlander E. et al | NA | Abdominal pain  Pelvic pain  Nausea  Vomiting  Change in bowel habit  Melena  Anemia | Other diseases of anus and rectum (e.g., haemorrhoids and perianal venous thrombosis, benign neoplasm)  Unspecified noninfective gastroenteritis and colitis  Other soft tissue disorders  Hypertension | NA | NA | NA | 184/361 |
| Oliver A.S. et al | Age  Gender | Yellow fingers  Anxiety  Peer pressure  Fatigue  Wheezing  Coughing  Dyspnea  Swallowing difficulty  Chest pain | Chronic diseases  Allergy | NA | Smoking status  Drinking status | NA | 15/15 |
| Rajan et al | NA | Dyspnea  Wheezing  Swallowing difficulties  Clubbing of finger nails  Frequent cold  Dry cough  Snoring | NA | NA | NA | Imaging test  Biopsy | NA |
| Senturk Z.K. et al | Age  Gender  City | Duration of symptoms Dyspnea  Chest pain  Weakness | NA | NA | Smoking status | Asbestos exposure  Diagnosis  Imaging test  Blood test  Performance status  Pleural Fluid Test  Dead or not | 34/34 |
| Wani N.A. et al | Age | Dyspnea  Chest pain  Coughing  Wheezing  Swallowing difficulty  Yellow fingers  Fatigue | Chronic diseases Allergy  Anxiety | NA | Smoking status  Drinking status | Peer Pressure | 16/16 |
| Weegar R. et al | Age | Cervical dysplasia (CIN 3)  Bleeding (postmenopausal) | Other cancer of female genital organs  Unspecified cancer of corpus uteri | NA | NA | Laboratory test  Medical procedures | 75000 |
| Xie S. et al | Age  Gender  Education level  Married or cohabiting | Reflux symptomps  Esophagitis | BMI | NA | Smoking status | Diaphragmatic hernia, Gastrointestinal surgical history | 8/NA |
| Zadsafar F. et al | Age  Gender  City | Chest pain  Weakness | NA | NA | Smoking status | Asbestos exposure  Type of mesothelioma  Pleural Fluid Test  Blood test  Biomarkes | 17/34 |

**Supplementary Table 4. Risk predictors: specific variables included in “other”.**

| **Authors** | **Other*** | **Other completed** |
| --- | --- | --- |
| Alam T.M. et al | Asbestos exposure Clinical data  Blood cell count  Clinical biochemistry test  Pleural data | Asbestos exposure  Duration of asbestos exposure  Lung side  Performance status  WBC  HGB  PLT  Pleural fluid WBC count  Erythrocyte sedimentation rate  Pleural to serum LDH ratio  Pleural to serum protein ratio  Albumin gradient  Blood glucose  Pleural glucose  Pleural effusion  Pleural thickness on tomography  Pleural level of acidity (pH)  CRP |
| Al-Juboori F.S. et al | Cancer test  Cancer stage and type  Receptor status  Cell activity  Treatment and Condition | Oncotype DX test  Stage of cancer  ER+  PG +  EGFR +  Proliferative activity of cells  Operation removal of the tumor or breast  Advanced type of tumor  Lymph nodes  Metastasis |
| Bhuta P. et al | Type of treatment  Origin of tumor | Treatments (Radiation, Medications, Surgery)  Origin of tumor |
| Briggs E. et al | Blood count cell  Clinical biochemistry test | Blood count cell (low MCV, high PLT, low HGB, high IM, high WBC))  Clinical biochemistry test (high LFT, cholesterol) |
| Chen A. et al | NA | NA |
| Chen J.H. et al | Imaging tests  Breast health evaluations and procedures | Imaging test of breast (ultrasound, MRI, mammogram, lab test)  Lab test or breast tumor removal surgery  Underarm examination or breast augmentation surgery |
| Chen J.W. et al | Serum marker  Head and neck mass management | Serum marker  Head and neck mass management |
| Chen Q. et al | Pancreatic disorders (not diabetes)  Medication  Outpatient visits and diagnostic test | Pancreatic disorders (not diabetes)  Use of metformin HCL, use of ciprofloxacin  Other screening for suspected conditions  Level 3, outpatient visit (established patients)  Level 4 outpatient visit (new patients)  Level 5 outpatient visit (established patients)  Measurement of lipase  Direct measurement of high-density cholesterol  Acute hepatitis panel  Residual codes—unclassified |
| Chicco D. et al | Asbestos exposure  Blood test  Pleural data and fluid test  Imaging and functional test  Dead or not  Type of malignant Mesothelioma  Performance status | Lung side  PLT  Pleural protein  Duration of asbestos exposure  CRP  Pleural albumin  Sedimentation  Glucose  Albumin  Pleural lactic dehydrogenise  Type of malignant mesothelioma  Asbestos exposure  WBC  Cytology exam of pleural test  Pleural thickness on tomography  HBG normality test  Performance status  LDH  Pleural effusion  Dead or not  Pleural level of acidity  Alkaline phosphatise  Total protein  Ache on chest  Pleural fluid WBC count |
| Choudhury A. et al | Asbestos exposure  Blood count cell  Clinical biochemistry test | Asbestos exposure  Duration of asbestos exposure  WBC  HGB  PLT  LDH  ALP  Total protein  Albumin  Glucose  Pleural lactic dehydrogenizes  Pleural protein  Pleural albumin  Pleural glucose  Pleural effusion  Ph  CRP |
| Dirik M. et al | NA | NA |
| Duan S. et al | Biomarkers  Radiomic features | Biomarkers: ProGRP, VEGF, CEA, CYFRA21-1, and NSE  Radiomic features: 22 CT nodule-based radiomic features |
| Erdemoglu E. et al | Diagnostic procedures  Surgical procedures | Endometrial biopsy  Dilation and curettage (D&C)  Hysterectomy  History of breast cancer |
| Goryński K. et al | Blood test  Pulmonary Function test  Imaging test | RBC  Erythrocite sedimentation rate  FEV1  Forced vital capacity (fvcex)  HT  HBG  WBC  X ray  PLT  Aspat (glutamic oxoloacetic transaminase)  Fev1/fvc-fev1-percent (fev1%)  VC  Creatinine |
| Hossain M.A. et al | NA | NA |
| Hu X. et al | Asbestos exposure  Diagnostic information  Blood test  Pleural fluid test | Duration of asbestos exposure  Diagnosis method  Cytology  WBC  HBG  Blood lactose dehydrogenase level  Albumin level  Glucose level  Pleural lactose dehydrogenase level  Pleural protein level  Pleural glucose level  Cell count  Pleural level of acidity |
| Kinar Y. et al | Blood test | CBC |
| Lindvall C. et al | NA | NA |
| Lo C.L. et al | Cystoscopy | Cystoscopy |
| Malhotra A. et al | Vascular conditions  Liver and gallbladder conditions  Gastrointestinal conditions  Drug-Related Issues  Kidney and Urinary Conditions  Gynecological conditions  Atopic diseases | Migratory thrombophlebitis  Jaundice  Gastrointestinal conditions  Oesophago-gastric problems  Irritable bowel syndrome  Diverticular disease  Pancreatitis  Abdominal mass  Gallbladder diseases  Oral problems  Inflammatory bowel disease  Steatorrhoea  Drugs  Kidney problems  Urinary problems  Gynecological conditions  Endometriosis  Fibroids  Atopic diseases |
| Masadah R. et al | Abdominal enlargement  Biomarkers | Abdominal Enlargement  % Beta Catenin/H-Score Beta Catenin |
| Mezher M.A et al | NA | NA |
| Mishra S. et al | NA | NA |
| Muhammad W. et al | NA | NA |
| Mukherjee S. et al | Asbestos exposure  Diagnosis  Imaging and tests  Blood tests  Pleural fluid tests  Dead or not  Performance status | Asbestos exposure  Type of mesothelioma  Duration of asbestos exposure  Diagnosis method  Keep side  Cytology  Performance status  WBC  HGB  PLT  Sedimentation  LDH  ALP  Total protein  Albumin  Glucose  Pleural LDHs  Pleural protein  Pleural albumin  Pleural glucose  Dead or not  Pleural effusion  Pleural thickness on tomography  Pleural level of acidity (pH)  C-reactive protein (CRP)  Class of diagnosis |
| Nemlander E. et al | NA | NA |
| Nemlander E. et al | NA | NA |
| Oliver A.S. et al | NA | NA |
| Rajan et al | Imaging tests  Biopsy | Imaging techniques (CT scans, X-rays)  Biopsy |
| Senturk Z.K. et al | Asbestos exposure  Diagnosis  Imaging test  Blood test  Performance status  Pleural Fluid Tests  Dead or not | Asbestos exposure  Type of mesothelioma  Duration of asbestos exposure  Diagnosis method  Keep side  Cytology  Performance status  WBC  HGB  PLT  Sedimentation  LDHs  ALP  Total protein  Albumin  Glucose  Pleural LDHs  Pleural protein  Pleural albumin  Pleural glucose  Dead or not  Pleural effusion  Pleural thickness on tomography  Pleural level of acidity (pH)  C-reactive protein (CRP)  Class of diagnosis |
| Wani N.A. et al | Peer Pressure | Peer Pressure |
| Weegar R. et al | Laboratory test  Medical procedures | Laboratory test  Cervical conization  Biopsy  Colposcopy |
| Xie S. et al | Diaphragmatic hernia, Gastrointestinal surgical history | Diaphragmatic hernia  Previous surgery for gastric or duodenal ulcer |
| Zadsafar F. et al | Asbestos exposure  Type of mesothelioma  Pleural Fluid Tests  Blood tests  Biomarkes | Asbestos exposure  Duration of asbestos exposure  Lung side  Cytology  Type of mesothelioma  Exam of pleural fluid  Hemoglobin normality test  Pleural effusion  Pleural level of acidity  Pleural thickness  Albumin  ALP  CRP  LDH  Glucose  PLT  WBC  Pleural albumin  Pleural fluid WBC count  Pleural fluid glucose  Pleural lacticdehydrogenase  Pleural protein sedimentation rate  Total protein |

**Supplementary Table 5.** Classification of included studies according to the types of data used in machine learning models for cancer risk prediction. A study was classified as “Included” for a given data type if that type was explicitly used as input in the final model. Studies integrating three or more data types (excluding symptoms, which were present in all studies) are defined as multimodal and are shown in bold.

| **Study** | **Symptoms** | **Sociodemographic** | **Lifestyle** | **Comorbidities** | **Genetic/Lab** |
| --- | --- | --- | --- | --- | --- |
| Alam et al. | Included | Included | Included | Not included | Not included |
| Al-Juboori et al. | Included | Not included | Not included | Not included | Not included |
| Bhuta et al. | Included | Not included | Not included | Not included | Not included |
| Briggs et al. | Included | Included | Not included | Not included | Not included |
| Chen A. et al | Included | Included | Not included | Included | Not included |
| Chen J.H. et al | Included | Not included | Not included | Not included | Not included |
| **Chen J.W. et al** | Included | Included | Not included | Included | Included |
| **Chen Q. et al** | Included | Included | Included | Included | Included |
| Chicco et al | Included | Not included | Not included | Not included | Not included |
| Choudhury et al | Included | Included | Not included | Not included | Not included |
| Dirik et al | Included | Included | Included | Not included | Not included |
| Duan et al | Included | Included | Not included | Not included | Not included |
| Erdemoglu et al | Included | Included | Not included | Not included | Included |
| Goryński et al | Included | Not included | Not included | Not included | Not included |
| Hossain et al | Included | Included | Included | Not included | Not included |
| Hu et al | Included | Not included | Not included | Not included | Included |
| Kinar et al | Included | Included | Not included | Not included | Not included |
| Lindvall et al | Included | Included | Not included | Not included | Not included |
| Lo et al | Included | Included | Not included | Included | Not included |
| **Malhotra et al** | Included | Included | Included | Included | Not included |
| Masadah et al | Included | Included | Not included | Not included | Included |
| Mezher et al | Included | Not included | Included | Not included | Included |
| Mishra et al | Included | Included | Included | Not included | Not included |
| **Muhammad et al** | Included | Included | Included | Included | Included |
| Mukherjee et al | Included | Included | Included | Not included | Not included |
| Nemlander E. 2022 | Included | Included | Included | Included | Not included |
| **Nemlander E. 2023** | Included | Included | Not included | Included | Not included |
| Oliver et al | Included | Included | Not included | Included | Not included |
| Rajan et al | Included | Included | Not included | Not included | Not included |
| **Senturk et al** | Included | Included | Included | Not included | Not included |
| **Wani et al** | Included | Included | Included | Not included | Included |
| Weegar et al | Included | Included | Not included | Included | Included |
| Xie et al | Included | Included | Not included | Not included | Included |
| **Zadsafar et al** | Included | Included | Included | Not included | Included |
